# Enhanced Separation of Extracellular Vesicles Using Capillary Isotachophoresis With Spacer Compounds

**DOI:** 10.1002/elps.202400113

**Published:** 2025-04-06

**Authors:** Milan Pieter Paul de Putter, Andrea Capuano, Meia Numan, Thomas Hankemeier, Yuliya Shakalisava

**Affiliations:** ^1^ Metabolomics and Analytics Centre Leiden Academic Centre for Drug Research Leiden University Leiden the Netherlands; ^2^ EXIT071 B.V. Leiden the Netherlands

**Keywords:** capillary isotachophoresis, electrokinetic, extracellular vesicles, profiling, separation

## Abstract

Extracellular vesicles (EVs) are pivotal in numerous physiological and pathological processes, such as immune responses, viral pathogenesis, pregnancy, cardiovascular diseases, and cancer progression. Their capacity to influence complex intracellular pathways highlights their therapeutic potential in addressing various conditions, including neurodegenerative diseases and cancer. A novel capillary isotachophoresis (cITP) method was developed for the electrokinetic characterization of pre‐isolated EVs. Distinct peaks could be resolved at near‐baseline resolution using a novel mixture of spacer ions and laser‐induced fluorescence (LIF) detection. The vesicles were effectively separated from the unbound carboxyfluorescein diacetate succinimidyl ester (CFDA‐SE) amine‐reactive fluorescent stain used to detect them and from residual contaminants. The identity of the peaks shown in the electropherograms was validated via various methods, including incubation with specific antibodies or spiking of putative contaminants, such as proteins and lipoproteins. This report thus provides a detailed proof‐of‐concept for using cITP‐LIF for extracellular vesicle isolation, subtype fractionation, and profiling.

**Abbreviations**
EV(s): extracellular vesicle(s)
sEV(s): small extracellular vesicles(s)
lEV(s): large extracellular vesicle(s)
UC: ultracentrifugation
SEC: size‐exclusion chromatography
NVEPs: nonvesicular extracellular nanoparticles
TFF: tangential flow filtration
CE: capillary electrophoresis
ITP: isotachophoresis
cITP: capillary isotachophoresis
LIF: laser‐induced fluorescence
LE: leading electrolyte
TE: terminating electrolyte
CFDA‐SE: carboxyfluorescein diacetate succinimidyl ester
EOF: electroosmotic flow

## 1 Introduction

Extracellular vesicles (EVs) are nano‐sized, membrane‐bound structures secreted by cells into the extracellular space and ubiquitously released under both normal and pathological conditions [1]. These vesicles play a crucial role in intercellular communication and exhibit diverse contents, surface markers, and sizes, in the range of 30 nm (for small EVs—small extracellular vesicles [sEVs]) up to 1000 nm (for large EVs—large extracellular vesicles [lEVs]) [1, 2]. EV membranes and contents (e.g., proteins, RNA, DNA) reflect their cell of origin, making them valuable for cancer diagnosis [3, 4]. EVs are found in various biological fluids, making them ideal for liquid biopsy [5], particularly for hard‐to‐reach cells like those in bone marrow or beyond the blood–brain barrier [6].

Reliable isolation methods are crucial to fractionate different populations of EVs (e.g., with different physical characteristics or different origins) and to purify them from proteins, lipoproteins, cells, debris, and contaminants [7]. Standard techniques like differential ultracentrifugation (UC) and size‐exclusion chromatography (SEC) yield EV populations that do not reach the necessary level of purity for use in a clinical research setting [8]. They rely on size or density, making it difficult to remove particles with similar physical properties. Furthermore, conventional methods are also time‐consuming, labor‐intensive, and costly [9].

Immunoaffinity methods offer high purity and selectivity. However, extracting EVs from large‐scale samples may necessitate a significant amount of costly antibody reagents, encounter non‐specific binding, and provide a lower yield than size‐based separation methods [9]. Other methods, such as polymer precipitation and microfluidic‐based techniques, also suffer from a lack of purity [10, 11]. Different nonvesicular extracellular nanoparticles (NVEPs) are known to have a similar or identical size and density to EVs, including specific lipoproteins, supermeres, and the recently discovered exomeres [7]. Such confounding factors complicate analysis and may go unnoticed due to the lack of sophisticated characterization techniques adapted to EV analysis [12]. Particularly, lipoproteins have proven difficult to separate from EVs. This is mainly due to their greater abundance in human plasma, with a concentration between 20‐ and 100‐fold higher than that of circulating EVs [13, 14]. Minimal information for studies of extracellular vesicles (MISEV2023) guidelines (minimal information for studies of EVs) recommend combinational purification methods to enhance EV sample purity, especially for complex matrices like blood plasma [15]. For example, combining tangential flow filtration (TFF) or UC with SEC yields significantly more sEVs than UC alone, though it requires substantial buffer volumes, adding complexity, cost, and the risk of aggregate formation [16]. Although these combined methods improve purity and scalability, they can also increase processing time, potential sample loss, and costs.

Due to EVs’ negative surface charge, anion‐exchange chromatography effectively isolates EVs from protein contaminants and oppositely charged molecules, though complete lipoprotein separation remains challenging due to size similarities [17]. Additionally, tentacle‐type cation exchange resins can achieve over 90% EV capture efficiency [18], but variable cationic domains across EV types may impact yield and purity. Capillary electrophoresis (CE) is an analytical technique that relies on electrophoretic mobility and is performed in narrow‐bore fused silica capillaries, offering several advantages over the aforementioned conventional techniques, including relatively short run times, high automation and hyphenation potential [19], and low volume requirements (with injection volumes even in the nanoliter or picoliter range [20]), making it better suited for clinical applications. It is also recognized for its high‐resolution analytical potential [21]. CE has been applied to electrokinetic analysis and isolation of various particles, including viruses, bacteria, cells, and lipoproteins [22, 23, 24].

**FIGURE 1:**
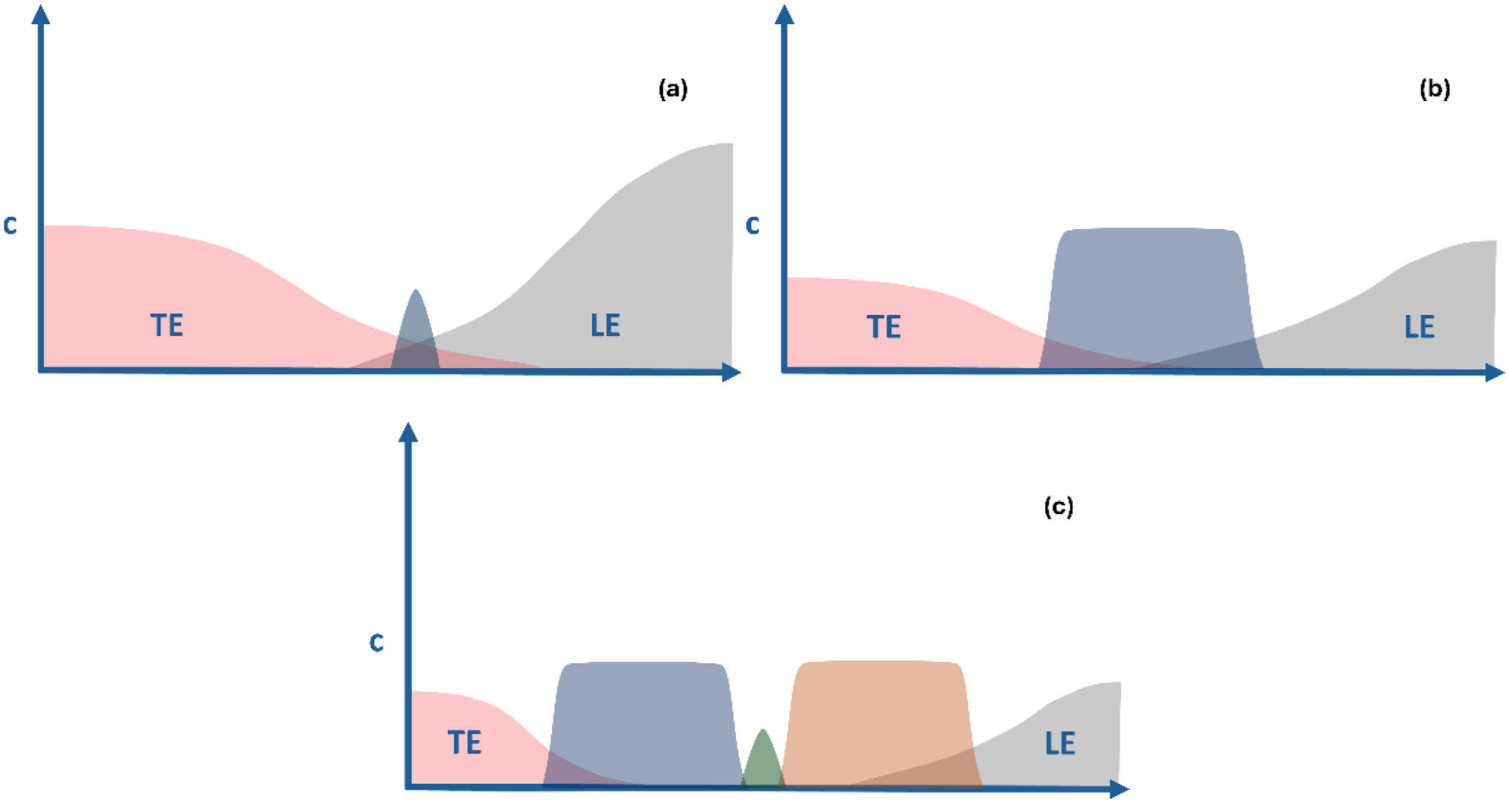
Schematic representation of the different distribution of analytes in peak‐mode (a) and plateau‐mode ITP (b). Once the concentration of a compound exceeds that of the LE and TE, it initiates plateau‐mode ITP, and the concentration within that zone remains constant. (c) Represents the case where a low abundant analyte is concentrated at the interface between two more abundant compounds that individually are in plateau mode (e.g., the spacer compounds), which is the mode that is exploited in this study. ITP, isotachophoresis; LE, leading electrolyte; TE, terminating electrolyte.

A specific type of CE called capillary isotachophoresis (cITP) has been applied to profile lipoproteins [25, 26, 27, 28]. In cITP, ions are separated in a discontinuous system, forming distinct zones between a leading electrolyte (LE) and a terminating electrolyte (TE) based on electrophoretic mobility. Applying constant electric current results in a steady state where ions move in constant, adjacent zones at a uniform speed determined by the LE [29]. The boundaries between these zones are self‐sharpening, as the electric field redirects diffusing ions back into their zone, granting cITP its extremely high‐resolution potential and preconcentrating power. In cITP, analytes exist in peak‐mode or plateau‐mode isotachophoresis (ITP) (Figure 1a,b). Plateau‐mode ITP occurs when a compound's concentration exceeds that of the LE and TE, leading to a constant concentration zone. Further increases in the amount of a plateau‐mode ion result in a widening of its zone as its concentration must be steady. Below this threshold, the analytes are in peak‐mode ITP. Analyte zones in peak‐mode ITP adapt their concentration to that of the LE as directed by the Kohlrausch regulating function [30]. Peak‐mode ITP zones thus experience a concentrating effect that makes ITP especially well‐suited for analyzing highly diluted trace analytes. Carefully selected spacer ions with specific electrophoretic mobilities can separate otherwise merged zones. Spacer compounds are ideally added in sufficiently high concentrations to trigger plateau‐mode ITP, thus forming broad zones. The actual trace analyte peaks, typically of significantly lower concentration than the LE/TE and thus in peak‐mode ITP, will focus instead on the sharp interfaces between two plateau‐mode peaks belonging to the spacer compounds or the LE and TE (Figure 1c) [31]. This process creates narrow, self‐sharpening fluorescent peaks of trace analytes between non‐fluorescent spacer zones, making cITP promising for EV subtype separation.

The use of cITP for preconcentrating EVs has been reported in a few studies [32, 33]. However, our investigation is the first to propose a strategy for preconcentrating and isolating EVs into distinct subgroups and enhancing the separation from contaminants by cITP through exploiting spacer compounds. Conversely, over the last decade, CE has seen various applications in EV characterization, employing diverse setups and methodologies. Piotrowska et al. [32] characterized EVs released by plant pathogens from the *Pectobacterium* genus with minimal sample consumption and rapid processing. Steć et al. [12] conducted a comparative analysis of EVs derived from *Pectobacterium zantedeschiae* culturing media, isolated using centrifugation‐based methods, assessing the selectivity of CE in comparison to techniques such as nanoparticle tracking analysis (NTA) and bicinchoninic acid assay (BCA). Morani et al. [34] used CE for the electrokinetic characterization of EVs from animal and human origins. The same group presented a Taylor dispersion analysis (TDA)‐based method [35] for absolute sizing of EVs without calibration. It also identified molecular contaminants (∼1–13 nm), enhancing EV isolate assessment. Dou et al. [36] introduced Extracellular Vesicles Quantitative Capillary Electrophoresis (EVqCE) to measure RNA mass within EVs and quantify EV concentrations and degradation levels after sample manipulation, such as sonication, storage, or freeze–thaw cycles. Ouahabi et al. [37] introduced a capillary electrophoresis‐ultraviolet diode array (CE‐UV‐DAD) technique to profile EV preparations, minimizing EV adsorption onto capillary walls. Akagi and Ichiki [38] used a microcapillary chip‐based electrophoresis system for nanoparticle detection, facilitating detailed EV characterization. As with ITP, it is possible to simultaneously isolate and concentrate EVs using ion‐concentration‐polarization‐based (ICP) microfluidic chips. For instance, Marczak et al. [39] employed an ICP microfluidic device to isolate EVs from various matrices (e.g., cell culture media and blood serum), consistently capturing between 60% and 80% of EVs across all tested matrices. The electro‐driven methods described above have shown the ability to distinguish at most two groups attributable to EVs with limited baseline separation and control [12, 34]. This article introduces a novel strategy for rapid, controllable fractionation of distinct EV subpopulations using cITP and fine‐tuned spacers. Moreover, this method promises significant utility in clinical research and EV manufacturing by enhancing the separation of EVs from contaminants left after conventional purification. This also makes our strategy promising for preparative applications. Several methods confirmed peak identities, such as CD81 and CD42b antibody assays and albumin, HDL, and VLDL lipoprotein spiking.

## 2 Materials and Methods

### 2.1 Chemicals

The chemicals and spacers used in this research were obtained from the following manufacturers: 2‐amino‐2‐methyl‐1,3‐propanediol (ammediol): A9754 SIGMA, Triton X‐100: SIGMA 28210, 3l‐Ala: SIGMA G5129, l‐alanyl‐l‐alanine (AlaAla): SIGMA A9502, 1‐methyl‐l‐histidine: Aldrich 67520, 3‐methyl‐l‐histidine: SIGMA M9005, l‐Cys, l‐Ser, l‐Val, l‐Met, l‐Gln, l‐Gly, l‐Glu, l‐Phe: FLUKA 21‐L‐amino acids + Glycine kit SIGMA‐Aldrich prod. 09416, l‐alanyl‐glycine (AlaGly): SIGMA A0878, glycyl‐l‐histidine (GlyHis): SIGMA G1627, glycyl‐l‐phenylalanine (GlyPhe): SIGMA G2752, 2‐[*N*‐morpholino]ethanesulfonic acid (MES): SIGMA M8250, d‐glucuronic acid: Alfa Aesar L14350, 3‐morpholino‐2‐hydroxypropanesulfonic acid (MOPSO): SIGMA M8389, Octanesulfonic acid sodium salt: SIGMA O8380, *N*‐[tris(hydroxymethyl) methyl]‐3‐aminopropanesulfonic acid (TAPS): SIGMA T5230, *N*‐[tris(hydroxymethyl)methyl]‐3‐amino‐2‐hydroxy propanesulfonic acid (TAPSO): SIGMA T9269, Tricine: SIGMA T0377.

### 2.2 Spacer Compound Mixtures

The initial spacer mix used to conduct this study consisted of 24 compounds. The electrophoretic mobility of these compounds within the system was initially predicted using the PeakMaster software [40]. This original 24‐spacer mix was adapted from [25], where the cITP fractionation of serum lipoproteins is achieved using ionic spacer compounds. Given that lipoproteins share certain physical characteristics with EVs—such as nanoscale size and similar density [41]—we hypothesized that selecting spacers within the same electrophoretic mobility window reported in [25] would allow for the effective fractionation of EVs. Subsequently, the spacer mixes were refined, keeping only the compounds with a mobility range compatible with the position (migration time) of the EV‐related peaks in the electropherogram. The electrophoretic mobility of each spacer compound was assessed by conducting a series of individual spikes of each spacer compound at concentrations 0.32 and 2.1 mg/mL and observing their effects on the electropherogram, as detailed in Section 3.1. Zohouri et al. [33] reported electrophoretic mobility for EVs in the range of −5 to −9 × 10^−9^ m^2^/s/V, whereas Kato et al. [42] reported a broader range of −5 to −15 × 10^−9^ m^2^/s/V, depending on the cell of origin. The final spacer combination used in our study covers a wide mobility range, from −5.7 (TE mobility at pH 9.2) to −25.7 × 10^−9^ m^2^/s/V (MES spacer compound mobility at pH 9.2). On the basis of our measurements and the peak identification attempts (Sections 3.3–3.7), the mobility window for EVs in this study is between the mobility of TE (−5.7 × 10^−9^ m^2^/s/V) and the spacer compound serine (−14.4 × 10^−9^ m^2^/s/V). Beyond this point (toward lower migration time), the electropherogram peaks indicate at least partial mixing of EVs with contaminants (Sections 3.3–3.7). During the study, different mixes were developed, and different concentrations of compounds were tested to optimize separation. Moreover, some compounds were replaced with others to test different separation windows. In this way, four different spacer compositions (named MIX A, B, C, and D) were obtained and reported. The compositions of spacer compound mixtures used for the different experiments and the final concentration of each compound in the final mix in the tested EV samples are given in Table S1.

### 2.3 EV and Carboxyfluorescein Diacetate Succinimidyl Ester (CFDA‐SE) Sample Preparation

Lyophilized enriched HEK293 cell‐derived (HBM‐HEK293), SK‐N‐SH neuroblastoma‐derived (HBM‐SK), and plasma‐derived EVs (HBM‐PEP) were purchased from HansaBioMed Life Sciences (Tallinn, Estonia). The EV samples were purified by HansaBioMed Life Sciences using a protocol combining UC and microfiltration steps. Before use, the EV samples were reconstituted in deionized water to a concentration of 1 mg/mL by pipetting up and down 15 times, avoiding the formation of bubbles. The resuspended standard was then vortexed for 60 s. To confirm the integrity and size distribution of the vesicles used in this study, we performed a preliminary analysis using TEM and dynamic light scattering (DLS), available in Figures S1 and S2. To visualize EVs for fluorescent detection, CFDA‐SE dye (5(6)‐CFDA‐SE—Thermofisher, Vybrant CFDA‐SE Cell Tracer Kit, V12883) was used, which can fluorescently stain EVs without altering their light‐scattering pattern [43]. Once inside the vesicles, CFDA‐SE is cleaved by esterases, forming a highly fluorescent green carboxyfluorescein succinimidyl ester. The ester then reacts with amines inside the vesicles, preventing it from passing through the lipid membrane. Nevertheless, unconjugated compounds and by‐products can diffuse out of the vesicle [43]. A 10 mM stock solution of CFDA‐SE was prepared by dissolving the content in DMSO, freezing it at −20°C, and guarding it against light. Shortly before each experiment, the CFDA‐SE stock solution was diluted to the desired concentration with PBS (40 µM in the final mix with EVs unless otherwise indicated). CFDA‐SE was then added to the sample in a 1:1 volume ratio. The protocol was adapted from [25]. The sample was subsequently incubated at 37°C for 2 h. After incubation, the sample was transferred to a plastic CE sample tube, after which the spacer mixture was added. All the experiments were performed with LoBind 1.5 mL Eppendorf tubes. The investigated volume for each experiment was 20 µL. For the UC experiments (Section 3.7), a Beckman Airfuge Compact Ultracentrifuge with rotor has been used. The ultracentrifuged sample volume was 0.5 mL.

### 2.4 cITP‐LEDIF

The LE was prepared with 10 mM HCl and 0.35% hydroxypropylmethylcellulose (HPMC), and the TE consisted of 20 mM l‐alanine (l‐Ala), both adjusted with 2‐amino‐2‐methyl‐1,3‐propanediol (ammediol) to, respectively, pH 8.8 and pH 9.4. An Agilent 7100 CE system was connected with a Zetalif 480 nm LED‐induced fluorescence detector for cITP‐LEDIF analysis. Prior to using our laser‐induced fluorescence (LIF)‐based method, we attempted detection with UV. In fact, fluorescent labeling may interfere with downstream applications and analyses. For instance, if the labeled EVs are intended for functional studies or therapeutic applications, the dyes could alter their biological activity or cellular uptake [44]. However, in the context of our cITP‐LIF method, fluorescent labeling was necessary to achieve the required detection sensitivity. Indeed, the UV signal was unsatisfactory, likely due to the relatively low particle concentration of 0.25 × 10^9/mL (e.g., 0.1 mg/mL of EVs diluted from the original stock). The challenge of detecting such low numbers of EVs using UV is consistent with difficulties reported in the literature for capillary electrophoresis‐ultraviolet (CE‐UV) [34]. OV1701‐OH coated fused silica capillaries (BGB Analytik AG, Switzerland) with an internal diameter of 100 µm, total length of 32 cm, and effective length of 20 cm were used. A preconditioning wash was performed by flushing with LE for 10 min when a new capillary was inserted into the machine. The capillary was flushed for 120 s with LE before each injection. The samples were injected by applying −50 mbar of pressure in the sample vial for 42 s. A voltage of 9 kV was applied across the system for 15–25 min, depending on the experiment, to leave enough time for all analytes to leave the capillary. After each run, the capillary was post‐conditioned with a flush consisting of consecutively 90 s of LE, 24 s of deionized water, 60 s of 1.5% Triton X‐100, 40 s of deionized water, and a final 120 s of LE. Each run was carried out in triplicate to assess the repeatability of the results. The software OpenLab CDS (ChemStation Edition—2019) was used to operate the system. The area under the electropherogram peaks was calculated with a built‐in function that automatically removed the background signal to have a repeatable baseline.

### 2.5 TEM

A copper mesh grid (300 mesh, coated with a thin film of Formvar—SIGMA ALDRICH 930253) was used to prepare EV samples for TEM. First, a 1% vol glutaraldehyde fixing solution (in water) was prepared. A layer of parafilm was placed over a dampened area on a clean work surface, and 5 µL of the EV solution was deposited onto the parafilm. Using tweezers, the grid was carefully positioned onto the EV solution drop. After 5 min, the excess liquid was gently removed from the grid using paper. The grid was then immersed in a drop of the fixing solution, and the excess solution was removed with paper. The grid was washed by placing it on a series of drops of distilled water while drying between washing steps using paper. Lastly, the grid was moved onto a drop of uranyl acetate for contrast staining, then dried with paper and stored in a grid box for subsequent analysis. JEOL JEM 1400 plus TEM was used for the analysis.

### 2.6 Dynamic Light Scattering

A DLS‐based Zetasizer Nano Series Model ZEN 3600 (Malvern Panalytical) was used to measure the particles’ size distribution (by volume). The Zetasizer software (2018) was used to run measurements and collect data. For the measurement process, 40 µL of the EV sample was used. The sample was loaded into the machine using a ZEN0040 40 µL cuvette.

### 2.7 CD81 and CD41b Antibody Assays

Two antibodies were chosen for an online antibody binding assay to investigate peak contents. CD81 was selected as a semi‐specific EV surface marker, whereas CD42b was selected for its presence on the surface of platelet EVs. The mouse anti‐CD81 (555675—0.5 mg/mL) and anti‐CD42b‐FITC (555472—size 100 tests) were obtained from BD Biosciences. For this group of experiments, the relevant antibody was first diluted five times using PBS 1x (from the original stock concentration). The diluted antibodies were then added to the EV sample in a 1:1 ratio, after which the sample was incubated for 30 min at 4°C. The samples were then stained with CFDA‐SE, as usual, before adding the spacer mixtures.

### 2.8 HDL and VLDL Lipoprotein Spiking

Two different lipoproteins, HDL (EMD Millipore Corp., 437641) and VLDL (EMD Millipore Corp., 437647), have been spiked into separate enriched plasma‐derived EVs. Each spike consisted of a volume of 5 µL: 0.16 mg/mL HDL or 0.145 mg/mL VLDL added to a volume of 20 µL (1:4 ratio) consisting of a 1:1 ratio of plasma‐derived EVs and 40 µM CFDA‐SE. As a control, one sample was spiked with 5 µL of PBSx1 instead.

## 3 Results and Discussion

### 3.1 Spacer Compounds Identification

**FIGURE 2:**
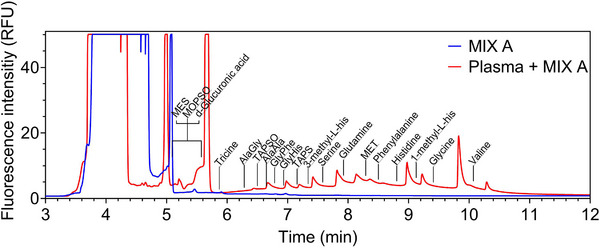
All the spacer compounds used for MIX A are shown in the electropherogram. The method is applied to a pre‐purified plasma sample (final concentration 0.1 mg/mL). The comparison with the blank (which contains only CFDA‐SE in the presence of the spacer mix) shows that the peaks related to the EV sample occupy a lower electrophoretic mobility zone than the peaks present when the sample contains only dye. The spacer compounds subdivide the analytes into subgroups.

We conducted a series of experiments to identify, starting from the initial 24‐compound mix, the ionic spacers with electrophoretic mobility closer to that of EVs. Figure 2 summarizes the method employed, where all spacer compounds are components of MIX A (Table S1). Specifically, a plasma EV sample, pre‐purified by the supplier using UC and microfiltration steps (see Materials and Methods), is depicted in the figure. As mentioned above, all the spacer compounds present in all the mixes reported in this study were spiked by using two different concentrations (0.32 and 2.1 mg/mL). In Figure S3, it exemplifies the method by showing an experiment involving one single compound, TAPS, using MIX A and a pre‐purified plasma‐derived EV sample (same conditions as Figure 2). The results indicate that increasing the concentration of a specific spacer widens the distance between consecutive peaks while preserving the number of peaks. Several experiments presented in this study show that the peaks in this region, characterized by lower electrophoretic mobility, are attributable to the presence of EVs. Therefore, spacers effectively divide the EVs in this region into subgroups. The use of specific spacers (as shown in Figure 2—MES, MOPSO, d‐glucuronic acid, and Tricine) enables the complete separation of EV sample‐related peaks from those observed when only CFDA‐SE is present (blank—MIX A). This is promising for sample preparation as it eliminates the need to clean EV samples of CFDA‐SE residues using molecular mass cutoff filters, which can damage EVs or reduce the overall yield of EV purification. Finally, as shown further in our findings, the peaks observed between the regions of the electropherogram associated with EVs and CFDA‐SE are attributable to contaminants that are fluorescently stained, such as lipoproteins (Section 3.6) and protein residues (Section 3.6 and Figure S7).

Because the number of spacers employed constrained these separations, it is conceivable that the actual count of separable groups within the cITP system could be even greater. In Figure S4, it shows the signal obtained with the same sample (plasma‐derived EVs) but in the presence of a different spacer mix containing fewer compounds (MIX D). In this case, fewer peaks and less differentiation between subgroups are observed. A signal in the absence of a spacer mix is also reported. In this case, the EVs coalesce into a single peak, which falls within the same mobility range as one of the CFDA‐SE peaks when it is present alone in solution.

### 3.2 EV Concentration‐Dependent Scaling of Fluorescence

**FIGURE 3:**
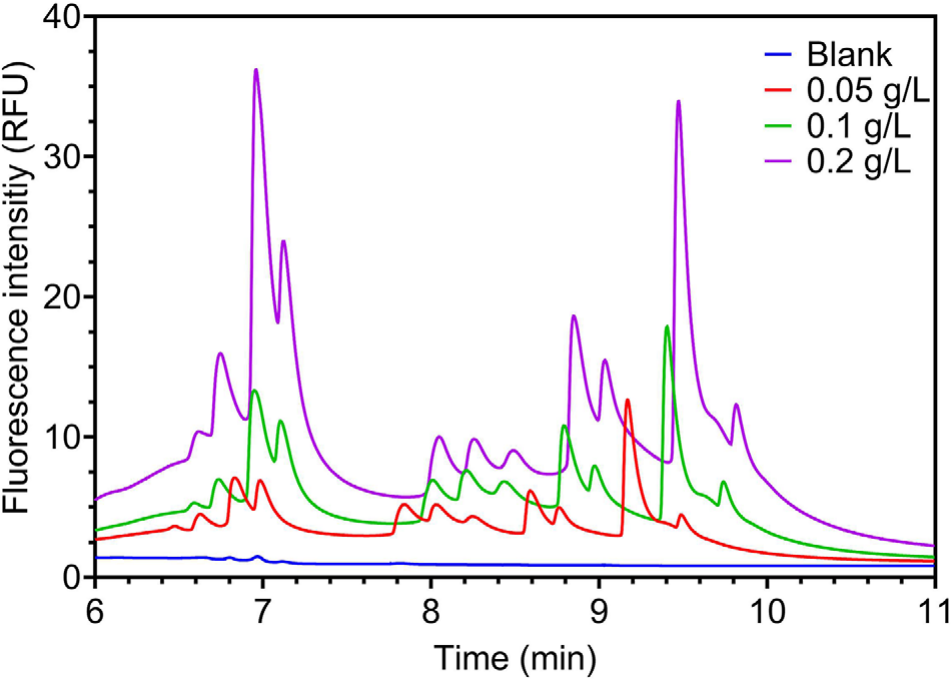
Zoomed‐in representation (6–11 min) of a cITP separation of three different concentrations of plasma‐derived EV samples stained with 40 µM CFDA‐SE. Blue: blank (MIX A, in the presence of CFDA‐SE, in PBS); red: 0.05 mg/mL; green: 0.1 mg/mL; and purple: 0.2 mg/mL. The signal intensity in this area of the electropherogram (lower mobility zone) increased with EV concentration.

In a series of experiments, the position range of EV‐associated peaks in the electropherogram was confirmed by using solutions with a constant CFDA‐SE concentration (40 µM) and varying plasma‐derived EV concentrations (blank, 0.05, 0.1, and 0.2 mg/mL). PBS buffer in solution with CFDA‐SE and MIX A was used as the control sample. Results reported in Figure 3 (minutes 6–11) indicated increased signal intensity at the tail end of the graph (lower mobility zone), correlating with EV concentration (the full electropherogram is reported in Figure S5). However, a satisfactory separation was not obtained because of the severe tailing effect of the peaks. Generally, this tailing effect can occur when a spacer ion has an electrophoretic mobility close to the bordering analyte ion [30]. Slight overlaps of individual ions cause dispersion of analytes between the zones, resulting in an increased diffused boundary scaling with the concentration of either the spacer or the charged species. Although coated capillaries were used in this study to eliminate the effect of electroosmotic flow (EOF), tailing may also occur because of residual EOF, which increases as the run progresses and can be further amplified by the increasingly large scale of the peaks [45, 46].

Besides the tailing effects, the results reported here confirm that in the zone between approximately 6 and 11 min, for the same concentration of spacer compounds and fluorescent dye, the signal depends solely on the concentration of pre‐purified EVs.

### 3.3 Signature Peak Patterns When Analyzing EVs From Different Origins

**FIGURE 4:**
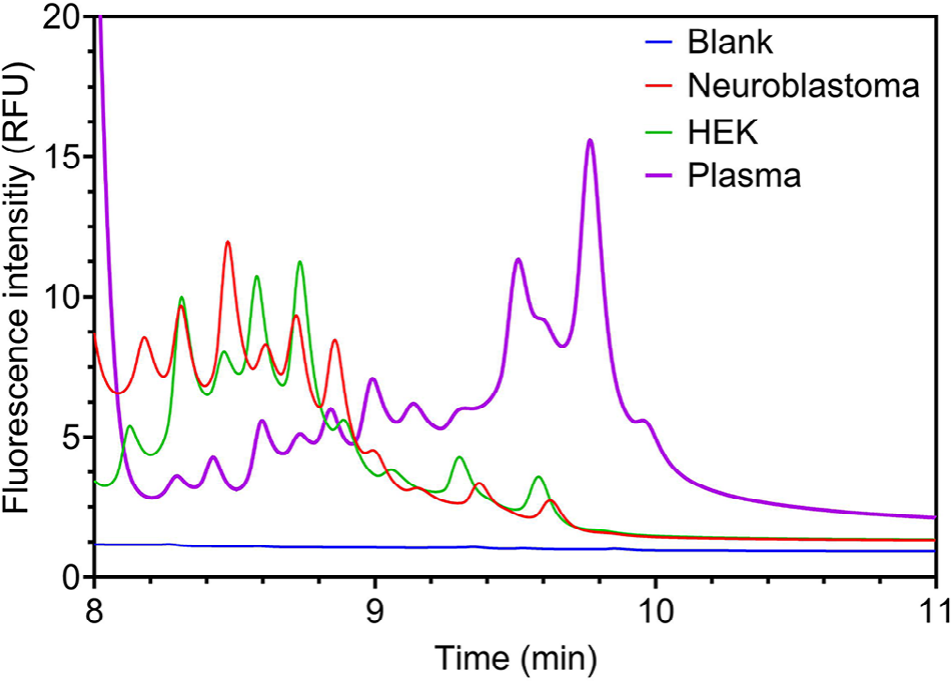
cITP separation of 0.1 mg/mL plasma‐derived EVs (green), HEK293 cell‐derived EVs (red), and SK‐N‐SH neuroblastoma‐derived EVs. Spacer mixture: MIX B. The method can detect distribution differences among EVs derived from various cellular origins.

In this section, we show the application of the same method, that is, separation of EVs in the presence of the spacer compound mix but comparing samples of different natures. In the electropherogram shown in Figure 4, EVs derived from HEK293 cell culture were compared to those isolated from plasma and those derived from SK‐N‐SH neuroblastoma cell lines.

Besides the source of EVs, all experimental conditions were identical. All volumes and concentrations in the tested solution were the same, including the concentration of enriched EVs (0.1 mg/mL) and fluorescent dye (40 µM CFDA‐SE). Upon analyzing the graphs, although CFDA‐SE‐associated peaks largely mirrored one another (the complete electropherogram is reported in Figure S6), variations in distribution were evident in the EV‐associated peaks. Each peak represents a cluster of analytes within a specific range of electrophoretic mobility dictated by the adjacent spacers. Consistent differences in distribution were observable across EVs from different origins. Plasma‐derived EVs exhibited a higher proportion of slow‐moving analytes, as evidenced by increased signals toward higher migration time (between 9 and 10 min), along with several peaks absent in the other samples. Conversely, EVs from HEK293 cells and SK‐N‐SH contained a relatively more significant fraction of analytes with higher electrophoretic mobility. The collection of fractions at fixed locations can shed light on potential differences in their content through subsequent omics analysis, such as mass spectrometry for lipidomics and proteomics or transcriptomics. Nonetheless, these findings underscore the technique's capability to detect distribution disparities among EVs derived from various cellular origins, carrying significant implications for its utility. Profiling differential expression rates of EVs from specific cell types or origins holds promise for diagnostic applications.

### 3.4 Online cITP CD81 Antibody Assay: Binding to EV‐Associated Analytes

**FIGURE 5:**
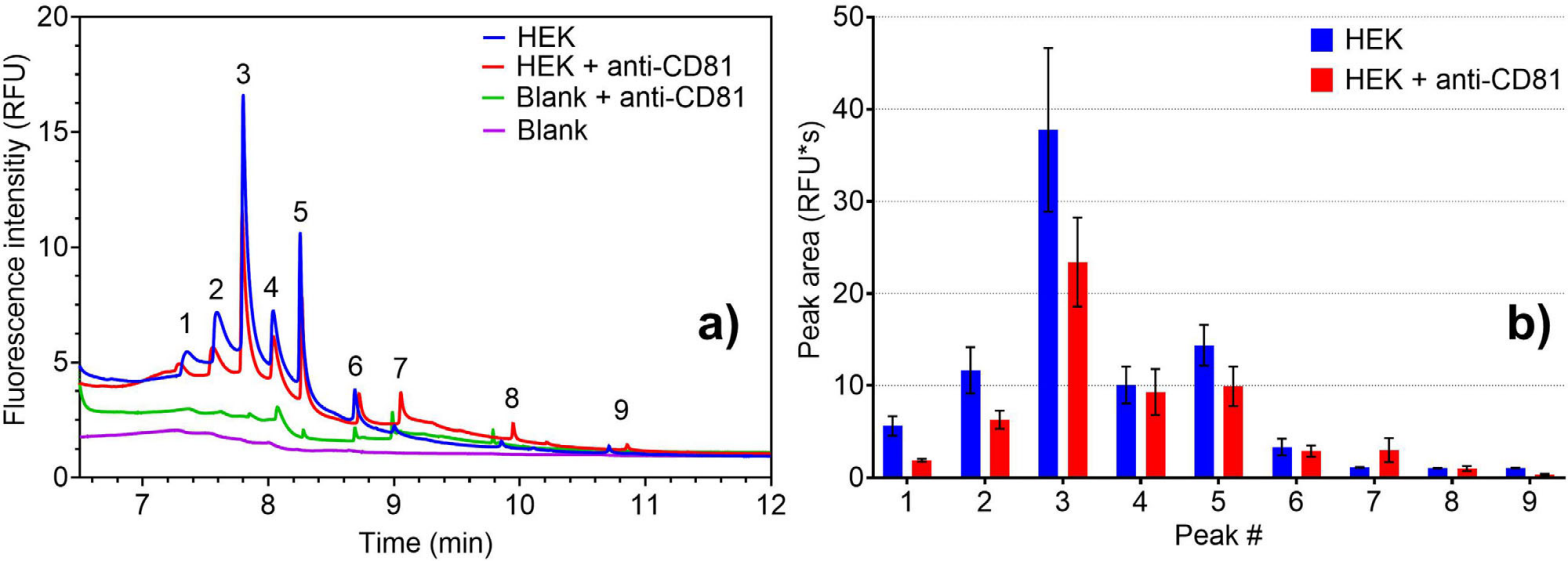
(a) Zoomed‐in view of a cITP separation of 0.1 mg/mL HEK293 cell‐derived EVs stained with 40 µM CFDA‐SE in the presence of anti‐CD81 antibody and control samples. Spacer mixture used: MIX C. Purple: no EVs/no anti‐CD81 (blank); green: no EVs (blank + anti‐CD81); blue: HEK EVs/no anti‐CD81; red: HEK EVs + anti‐CD81. (b) Average area of peaks 1–9 over three different measurements. The standard deviation is presented as an error bar for each peak. Adding the anti‐CD81 antibody to the EV sample decreased the peak intensity in at least four peaks between 7 and 9 min. For the other instances (e.g., later than 9 min), the antibody's fluorescence affected the signal more.

In this section, we report the use of affinity interactions to confirm the identity of EV peaks in our electropherograms. CD81 is a tetraspanin membrane protein known to be significantly enriched in the membrane of EVs [47]. The tests described in this section were performed after incubating HEK293 cell‐derived EV samples with the purified mouse anti‐human CD81 antibody, as described in the Materials and Methods section. This test aimed to assess whether the EV peaks were subject to a temporal shift in the electropherogram due to the electrophoretic mobility shift caused by the binding of CD81 to the specific antibodies. Figure 5 shows the results of the experiments. Due to the sufficient separation enhanced by the spacer mixture MIX C, peak areas could be integrated and compared (Figure 5b). The signal shows that the CD81 antibody was also stained by CFDA‐SE, resulting in several low‐intensity peaks overlapping with some EV peaks. Upon adding the CD81 antibody to the EV sample, peak intensity was significantly decreased in at least four EV‐associated peaks (between 7 and 9 min) compared to the sample without the antibody. Where the EV peaks were less intense (e.g., later than the 9‐min mark, peaks 6–9), the contribution of the antibodies’ fluorescence (blank + anti‐CD81—represented in green in Figure 5a) affected the final signal more. Indeed, for such peaks, the intensity in the combined sample could thus be attributed to the superimposition of the signals of both the unbound anti‐CD81 and the EVs.

In the provided electropherograms, the EV‐antibody complexes did not generate new peaks toward higher migration time (lower mobility) with respect to those present before the addition of antibodies. Consequently, the EV‐antibody complex showed increased electrophoretic mobility, and then the related peaks were expected to appear earlier in the electropherogram (in a higher mobility zone). Therefore, an explanation for the obtained graphs is that the EV‐antibody complex migrated to a mobility range close to that of CFDA‐SE (migration time earlier than 6.5 min), making it challenging to discern peak shifts due to peaks with high fluorescence intensity. Consequently, the observed decrease in peak area in the EV region of the electropherogram occurred because only a portion of the EVs bound to the antibodies, altering their mobility. This portion, which previously contributed to peak intensity before antibody addition, underwent a mobility shift, ending up in a region where it could not be distinguished from other high‐intensity peaks. Although electrophoretic mobility depends on a combination of size and charge, in the case of EV‐bound antibodies, the charge added by the antibodies (which are charged under the experimental conditions described) was a predominant factor and resulted in increased electrophoretic mobility of the EV‐antibody complex. In addition, it cannot be ruled out that the binding of antibodies to the EVs caused their partial precipitation before injection and, thus, a decrease in the observed fluorescence [48].

A similar experiment was then conducted using plasma‐derived EVs, yielding similar effects to those observed in the samples derived from HEK293 cells. Moreover, two different antibody dilutions (5 times diluted and 25 times diluted) were investigated in this experiment. The data are reported in Figure S8.

### 3.5 Online cITP CD42b‐FITC Platelet‐Associated Antibody Assay: Differences Between Plasma‐ and HEK293 Cell‐Derived EVs

**FIGURE 6:**
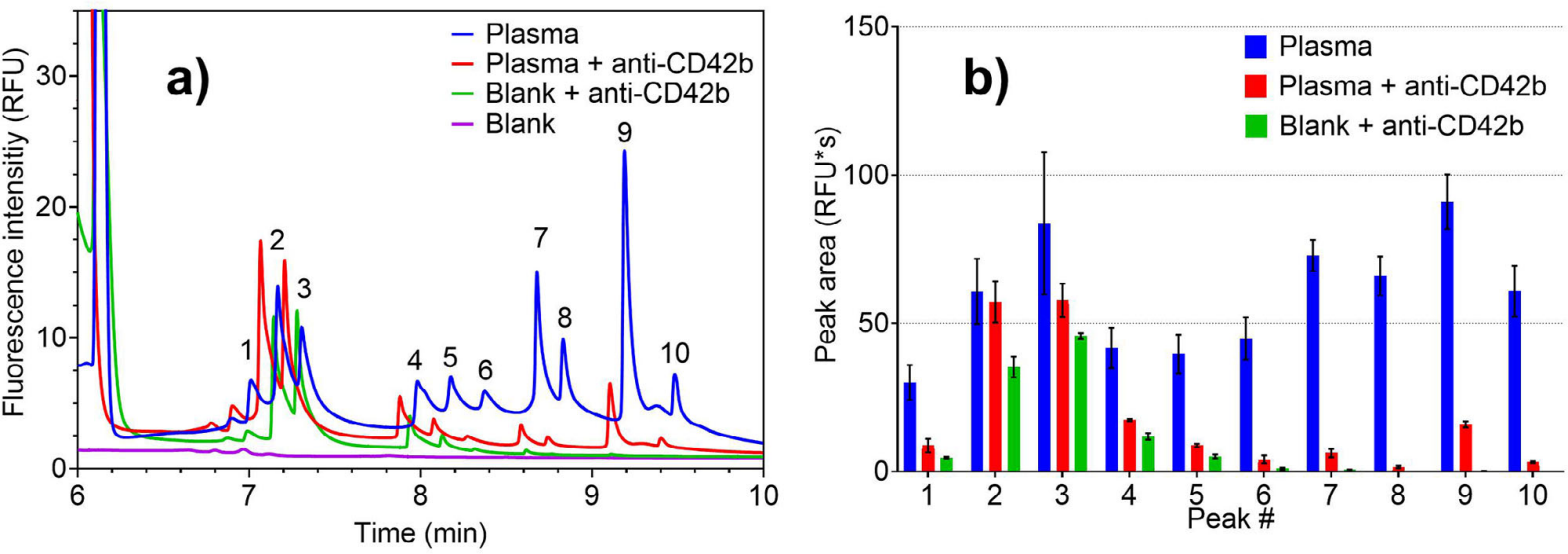
(a) Zoomed‐in view of a cITP separation of 0.1 mg/mL plasma‐derived EVs stained with 40 µM CFDA‐SE in the presence of anti‐CD42b antibody and control samples. Purple: no EVs (blank PBS1x in MIX B)/no anti‐CD42b; green: no EVs (PBS1x)/25x diluted anti‐CD42b; blue: plasma‐derived EVs/no anti‐CD42b; red: plasma‐derived EVs/25x diluted anti‐CD42b. (b) Average area of peaks 1–10 over three different measurements. The standard deviation is presented as an error bar for each peak. The anti‐CD42b antibody binding to platelet‐derived EVs impacts the peaks related to EVs, which have lower intensity.

CD42b is a protein expressed in platelets [49]. Plasma‐derived EV samples contain EVs originating from platelets, thus expressing CD42b. Conversely, HEK293 and most other cell culture‐derived samples are unlikely to contain this protein. By examining the effects of anti‐CD42b antibody binding on the EV‐associated peaks from both origins, more detailed conclusions can be drawn about the content of these peaks, especially when coupled with data obtained from the anti‐CD81 spike. Figure 6 presents an electropherogram illustrating the impact of anti‐CD42b‐FITC binding to plasma‐derived EVs. Reductions in most peaks were evident. The decrease observed between 8 and 9 min as the antibody is added to the sample is particularly noteworthy. However, the unequal baselines of the two samples introduce uncertainty into peak integration and subsequent analysis. This inconsistency could be attributed to inadequate separation in this region, compounded by tail‐end zone diffusion, leading to areas of signal overlap (as seen in Section 3.2). After conducting the initial experiment, the anti‐CD42b antibody assay was replicated using HEK293 cell‐derived EVs. Interestingly, although the superimposition of the fluorescent signal of the antibodies interferes with the evaluation of the peak area, it was evident that, in this case, there was no effect of decreasing peak intensity (Figure S9). The substantial decreases observed in the plasma‐derived EV sample but not in the cell‐derived EV sample are consistent with this experiment's hypothesis. The binding of the anti‐CD42b antibody to platelet‐derived EVs increases the electrophoretic mobility of the complexes, displacing them from their original positions. Conversely, cell‐derived EVs lacking CD42b remain unaffected. CD81 influenced both plasma‐derived and cell‐derived EVs, whereas only the former were affected by CD42b. Further experiments utilizing more specific antibodies could corroborate these findings and provide deeper insights into the precise contents of these peaks. The presented method is an initial proof‐of‐concept for simultaneous EV isolation and antibody‐mediated surface marker analysis. Enhancements in resolution, separation, and antibody assays could offer valuable insights into the nature of EVs present in a sample.

### 3.6 Purified Lipoprotein and Albumin Spiking

**FIGURE 7:**
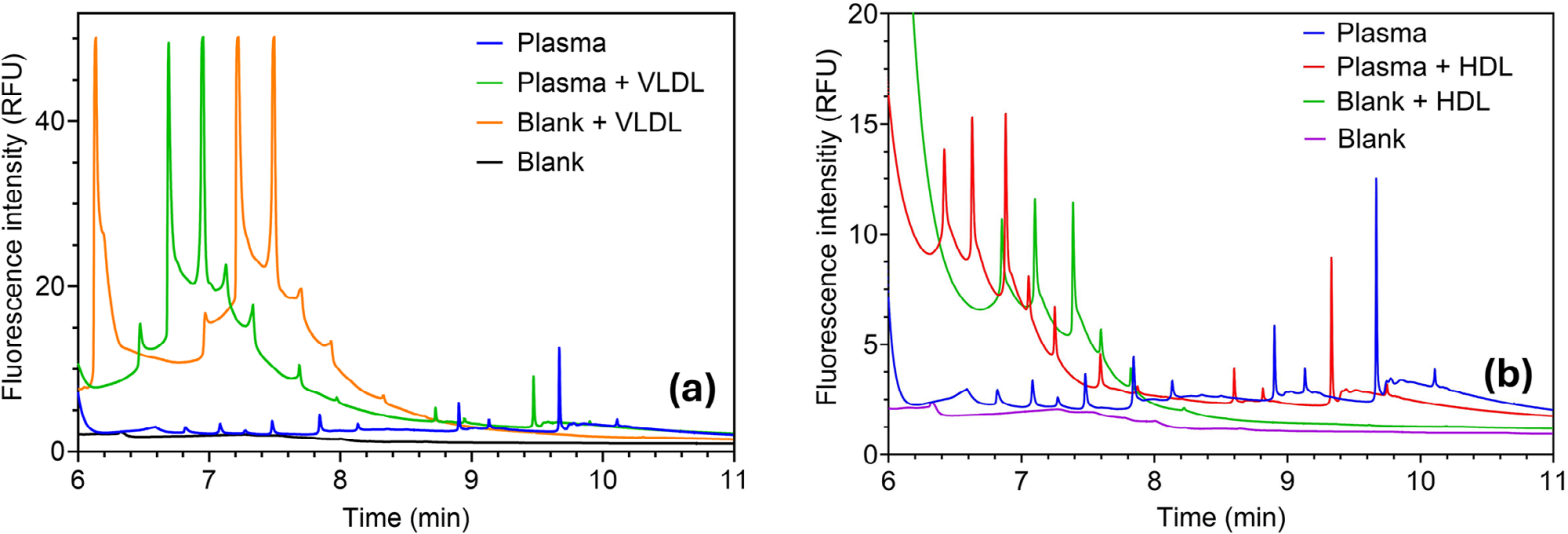
cITP separation of 0.1 mg/mL plasma‐derived EVs stained with 40 uM CFDA‐SE spiked with VLDL (a) and HDL (b). The blanks are also spiked with lipoproteins. Spacer mix used: MIX B. These findings suggest an overlap between lipoproteins and extracellular vesicles starting at mobility values around −14.4 × 10^−9^ m^2^/s/V (spacer compound serine—sooner than 8 min), whereas some peaks remain unaffected by added lipoprotein (in the lowest mobility area of the electropherogram).

As mentioned, lipoproteins have overlapping physical properties with EVs, making it difficult to separate them using conventional methods based on size or density. To investigate the position of lipoproteins in the electropherogram, a plasma‐derived EV sample was spiked with HDL and VLDL before staining with CFDA‐SE. The electropherograms reported in Figure 7 show the locations of lipoprotein spikes within the system. They typically emerge relatively early, between 5 and 6 min, showing higher electrophoretic mobility than EV‐associated peaks. Hence, these peaks are outside the EV‐associated range. Nonetheless, several peaks fall within the same range, albeit obscured by the tailing effect that elevates subsequent peaks following the spike‐induced peaks. In general, these findings suggest an overlap persists between lipoproteins and EVs within this system at mobility values above −14.4 × 10^−9^ m^2^/s/V (around the mobility of the spacer compound serine—refer to Table S1 and Figure 2), yet several peaks (in the lowest mobility area) remain unaffected by the added lipoprotein (e.g., around 8 min onwards). Hence, the current system necessitates a more precise spacer mixture to discriminate effectively between lipoproteins and all EVs. Notably, using size‐ and density‐based isolation methods to obtain enriched EV samples introduces the possibility that lipoprotein content persisted within the sample even before spiking. This is due to filtration's challenge in adequately separating lipoproteins from EVs due to their overlapping sizes.

Extracting the peaks and conducting a comprehensive analysis of their constituents is required to evaluate this hypothesis. In essence, the system demonstrates its capability to distinguish lipoprotein‐related peaks from EV‐related peaks. Although there is an overlap between small EVs with VLDL and HDL in size or density, this may not be the case for larger EVs. Through electrophoretic mobility‐based separation, it is conceivable that slightly larger EVs (e.g., 150 nm or higher) could be present in the peaks appearing later in the sample, whereas the smallest particles (e.g., ∼50–100 nm) may occupy the overlapping region.

In Figure S7, it is reported that albumin is also stained by CFDA‐SE and, thus, may be a contaminant in the processed EV samples. Protein residues may generally be present in plasma and samples derived from cell cultures. We show that the albumin mobility window lies between 6 and 7 min, which is intermediate between the CFDA‐SE and EV‐related peaks. Thus, our technique can separate this possible contaminant through the appropriate choice of spacers, with intermediate mobility between proteins and vesicles.

### 3.7 Effects of UC

**FIGURE 8:**
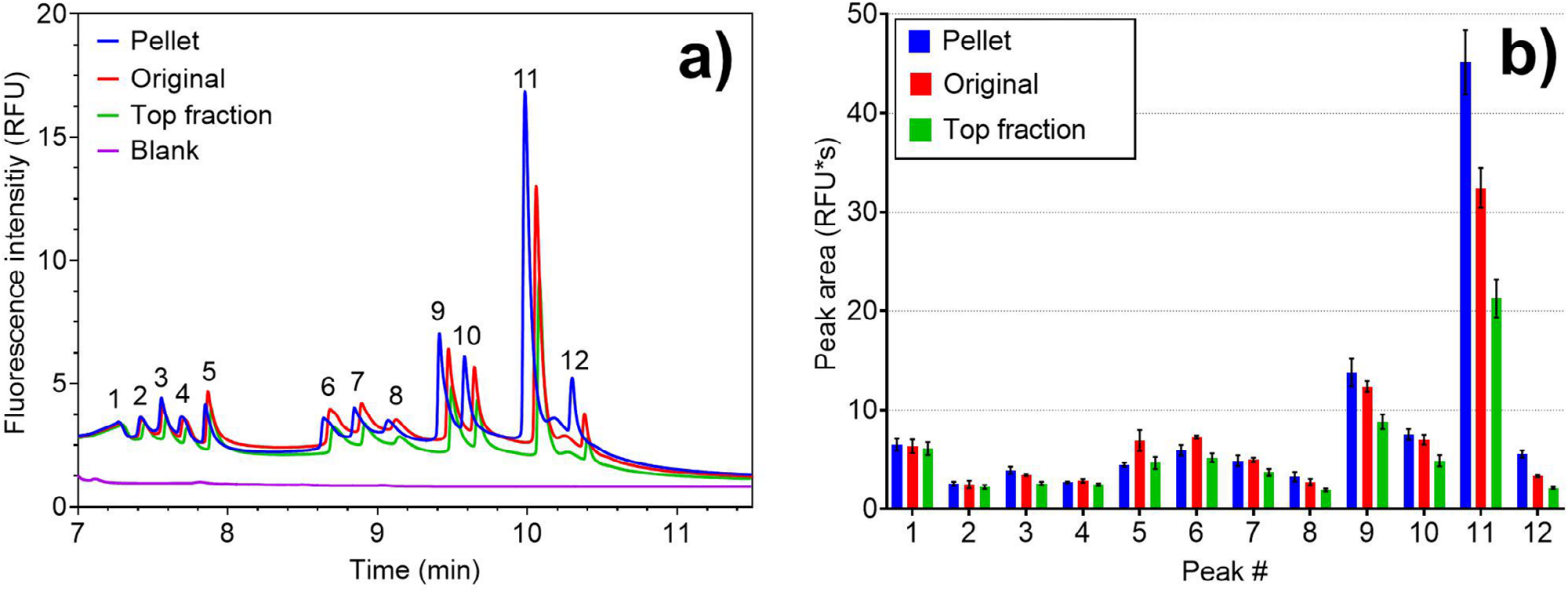
(a) Zoomed‐in view of a cITP separation of 0.5 mg/mL plasma‐derived EVs before (“original”) and after a 2 h centrifugation (“pellet” and “top fraction”) at 100 000 × g. (b) Average area of peaks 1–10 over three different measurements. The standard deviation is presented as an error bar for each peak. Spacer mixture used: MIX A. In the pellet, some analytes (e.g., peak 11) show up to 27% higher signal than the original sample and around 50% higher than the top fraction. Differences are minor for other peaks (e.g., 9, 10, and 12), whereas the remaining peaks show comparable intensities among pellet, original sample, and top fraction.

Although the samples analyzed for this study had already undergone purification protocols combining UC and microfiltration steps, some EV standards were again processed by UC (see 2.3 for details on the methods used). The effect on electropherograms was subsequently evaluated. As shown in Figure 8, the impact on some peaks is evident by ultracentrifuging 0.5 mL of the standard plasma EVs (0.5 mg/mL plasma‐derived, spacer mixture used: MIX A) at 100 000 × g for 2 h. In the pellet (highlighted in blue in the figures), specific analytes (e.g., peak 11) exhibit a signal up to 27% higher than the original sample and approximately 50% higher than the top fraction. Conversely, differences are comparatively minor for other peaks (e.g., 9, 10, and 12). For the remaining peaks, signal intensities are comparable among the pellet, original sample, and top fraction. The known impact of UC on EVs is to purify and concentrate them by sedimentation. This elucidates why concentration effects in the pellet are observed in some areas of the electropherograms, whereas signal intensities remain unchanged for other peaks following UC. It is hypothesized that the unaltered peaks in plasma may harbor contaminants or EVs of different natures and densities that are less affected by UC. Different UC protocols (e.g., longer time) could accentuate the difference in concentration between pellet and top fraction.

The experiment was replicated using the culture‐derived EV standard of HEK293 cell lines (0.5 mg/mL—Figure S10). In this instance, suboptimal peak separation, partially attributed to the aforementioned tailing effect, hindered accurate area calculations. Nevertheless, qualitative estimation reveals that, in this scenario, all peaks in the pellet exhibit higher intensities than both the original sample and the supernatant fraction, albeit with a slight temporal shift in the electropherogram. We can, therefore, hypothesize that the sample from cell culture exhibited greater purity than plasma, harboring a more significant number of peaks within the EV mobility zone whose intensities are impacted by UC.

## 4 Concluding Remarks

Our study demonstrates that cITP can enhance EV isolation by allowing precise control over separation using spacer compounds with defined electrophoretic mobility. As noted in the introduction, previous attempts using CE or cITP could not divide extracellular vesicle samples into multiple distinct subgroups characterized by unique peaks. We used pre‐purified EV standards at various concentrations, along with spiked contaminants, to characterize the electropherograms and observed distinct EV‐associated peaks. Incubating EVs with specific antibodies altered these peaks, confirming their identity and the method's specificity. A limitation of this study is the decision not to use non‐prepurified samples, such as raw plasma. This choice was made to show the technique's potential for characterizing vesicle samples and separating them into subgroups. Using raw samples would have introduced uncertainty in peak identification. However, this does not preclude the method's applicability in preparative contexts, particularly when combined with other approaches like SEC, UC, TFF, or anion‐exchange chromatography. In fact, comparing our approach to other methods in a sample preparation context was unfeasible for this study, as fraction collection was not performed. Future work could validate the method's feasibility for sample preparation to achieve clinical‐grade purity of EVs, particularly when combined with pre‐purification techniques recommended by the EV purification guidelines (MISEV 2023).

## Conflicts of Interest

The authors declare no conflicts of interest.

## Supporting information



Supporting Information

Supporting Information

Supporting Information

Supporting Information

## Data Availability

Data are openly available in a public repository that issues datasets with DOIs. The data that support the findings of this study are openly available in Data_DePutter_et al. at https://doi.org/10.5281/zenodo.11139247.
