# Supplementary material for: Enhanced Separation of Extracellular Vesicles Using Capillary Isotachophoresis With Spacer Compounds
Source: Electrophoresis. 2025 Apr 6;46(9-10):513–23. doi: 10.1002/elps.202400113 (PMC12273895; doi:10.1002/elps.202400113)
Supplement: Supplementary file 1 — Supporting Information [file ELPS-46--s002.docx]

Supporting information

Table S1. Spacer Mixture Compositions: Four different spacer mixtures (A, B, C, D) were utilized in this study. These mixtures were derived from an initial set of 24 spacers, selecting those that influenced peak spacing in the region associated with EVs (see Figure S1) and in the intermediate zone between the EV region and the one attributed to CFDA-SE (e.g., MES, MOPSO, D-glucuronic acid). The final concentration of each compound in the tested EV sample is detailed in the table.

| **Spacer Mix** | **Description** |
| --- | --- |
| **MIX A** | Concentration of spacers:  **MES, MOPSO, Serine, Tricine**: 1.667 mg/mL  **1-methyl-L-His, 3-methyl-L-His, AlaAla, AlaGly, D-glucuronic acid, Glycine, GlyHis, GlyPhe, Glutamine, Histidine, Methionine, Phenylalanine, TAPS, TAPSO, Valine**: 0.555 mg/mL |
| **MIX B** | Concentration of spacers:  **Glycine**: 0.448 mg/mL  **Glutamine, Methionine, TAPSO**: 0.716 mg/mL  **Serine**: 0.537 mg/mL  **TAPS**: 1.075 mg/mL  **1-methyl-L-His, 3-methyl-L-His, AlaAla, AlaGly, Cysteine, GlyGly, GlyHis, GlyPhe, Histidine, MOPSO, Phenylalanine, Tricine, Valine**: 0.600 mg/mL |
| **MIX C** | Concentration of spacers:  **1-methyl-L-His, 3-methyl-L-His, AlaAla, AlaGly, D-glucuronic acid, Glycine, GlyHis, GlyPhe, Glutamine, Histidine, MES, MOPSO, Phenylalanine, Serine, TAPS, TAPSO, Tricine, Valine**: 0.789 mg/mL |
| **MIX D** | Concentration of spacers:  **MES:** 2.344 mg/mL  **3-methyl-L-His, AlaAla, AlaGly, D-glucuronic acid, Glycine, GlyPhe, MOPSO, Serine, TAPS**: 1.406 mg/mL |


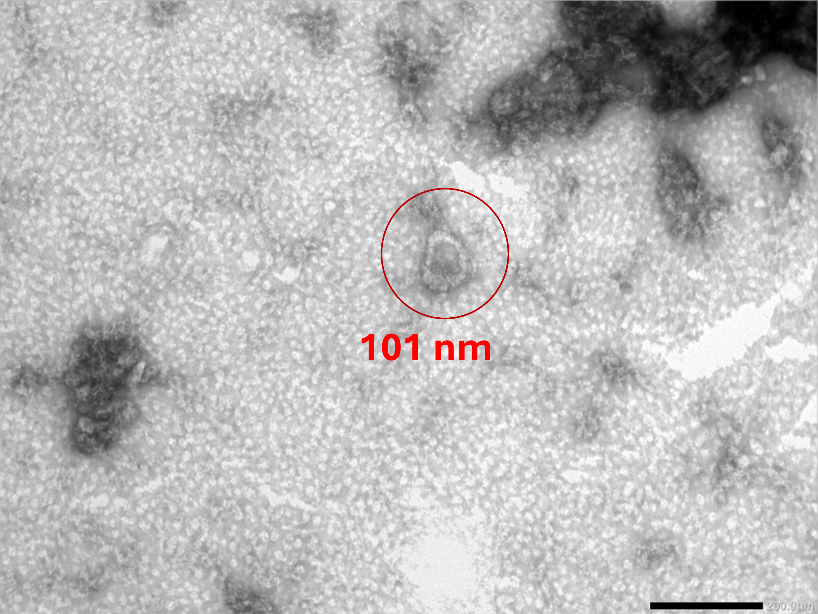

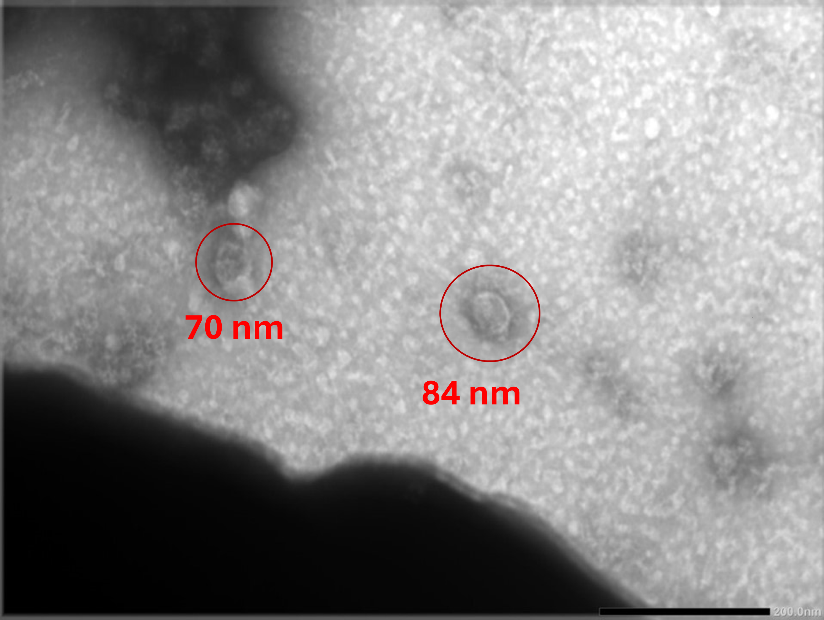

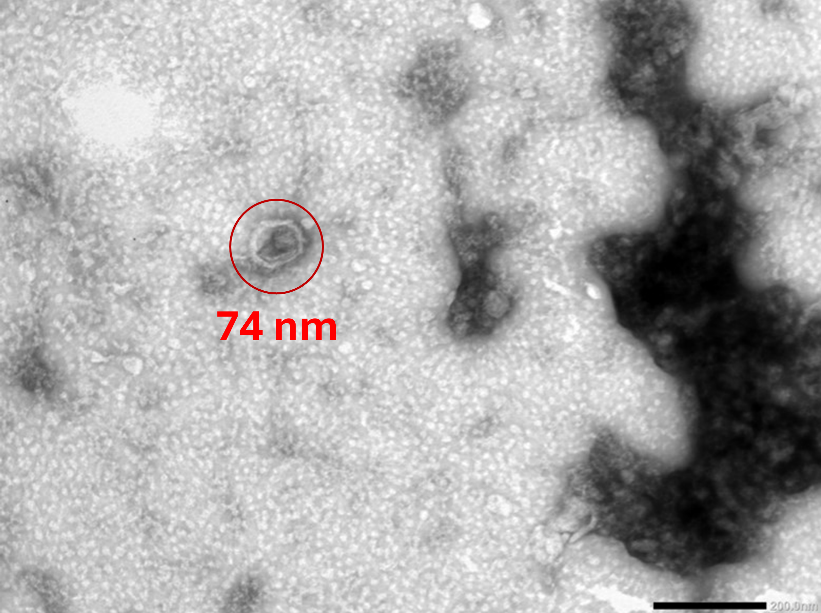


Figure S1. TEM images of HansaBiomed SK-N-SH Neuroblastoma-derived EVs (concentration 0.5 mg/ml – reference length 200 nm) used for the cITP experiments. Despite the lyophilization process, the integrity of the vesicles is preserved.


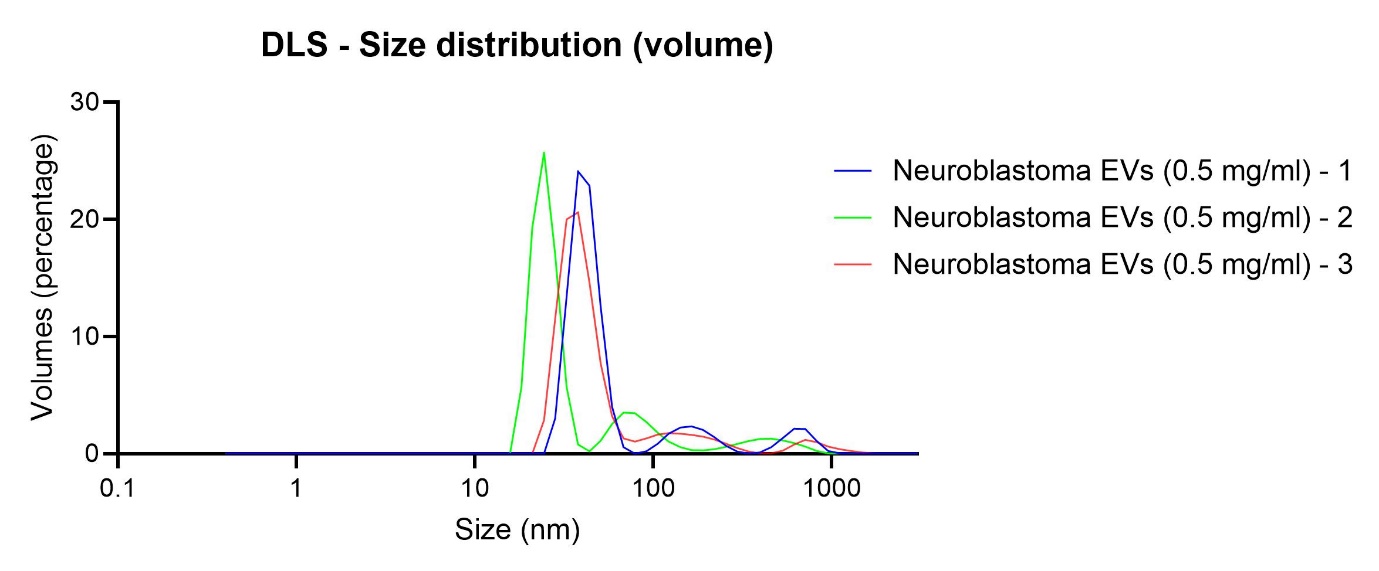


Figure S2. Dynamic light scattering (DLS) analysis of SK-N-SH neuroblastoma-derived EVs (HansaBiomed, concentration 0.5 mg/ml) used in the cITP experiments. Results from three measurements of the same sample show that most particles fall between 50 and 100 nm particle size, with a significantly smaller fraction outside this range. The residual presence of larger particles may be attributed to the purification protocol employed by the EV supplier. The results are consistent with the TEM inspection of the sample.


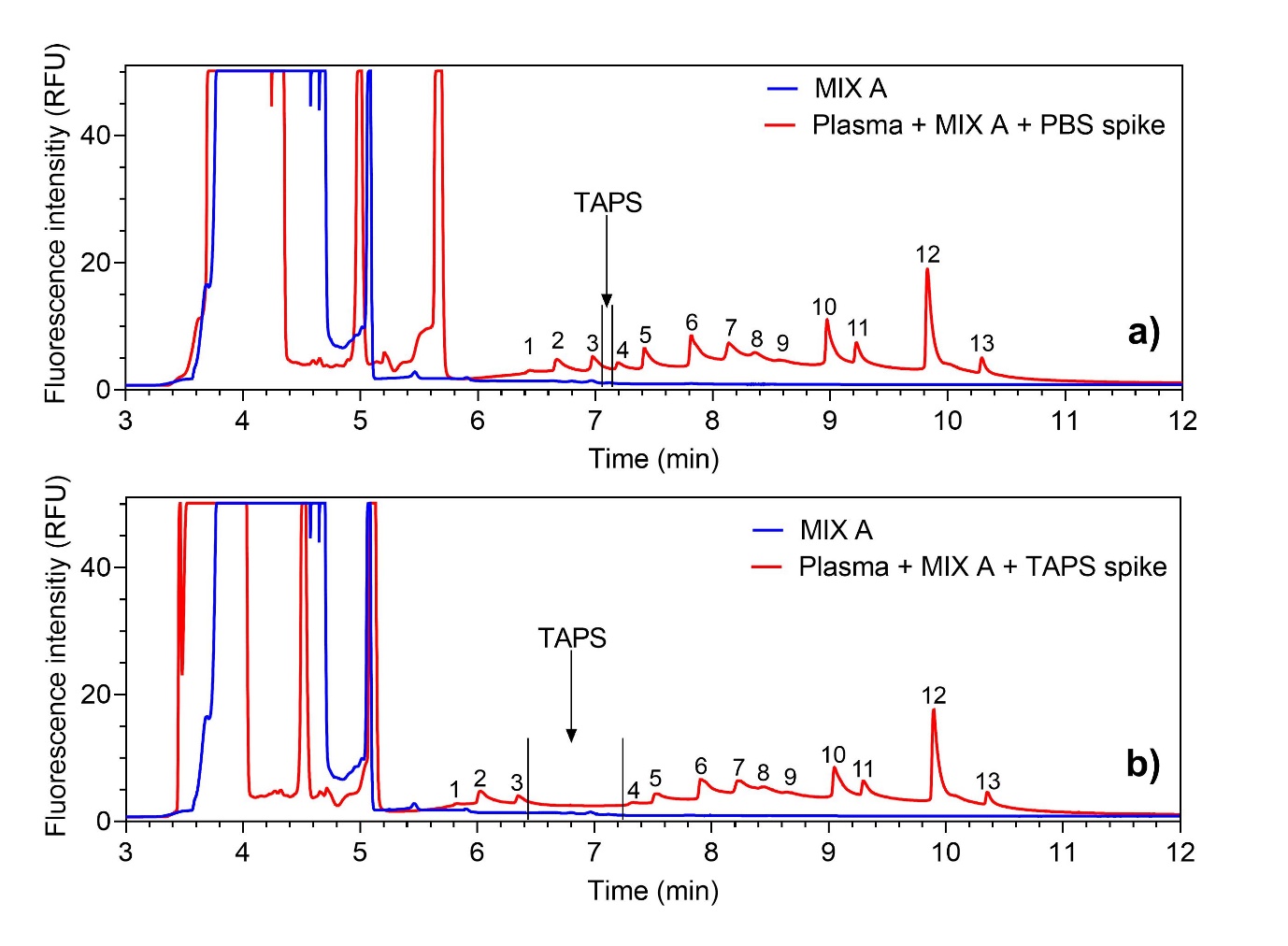


Figure S3. Experiment showing the effects of varying the concentration (0.32 mg/ml vs. 2.1 mg/ml) of a specific spacer compound, TAPS, using MIX A and a pre-purified plasma-derived EV sample under the same conditions as Figure 2. At the lower concentration, PBS is added to achieve the desired final volume and adjust the concentration of the spacer. The results demonstrate that increasing the spacer concentration widens the distance between consecutive peaks while preserving the number of peaks. Additionally, the experiment confirms that spacer compounds do not get stained by CFDA-SE.


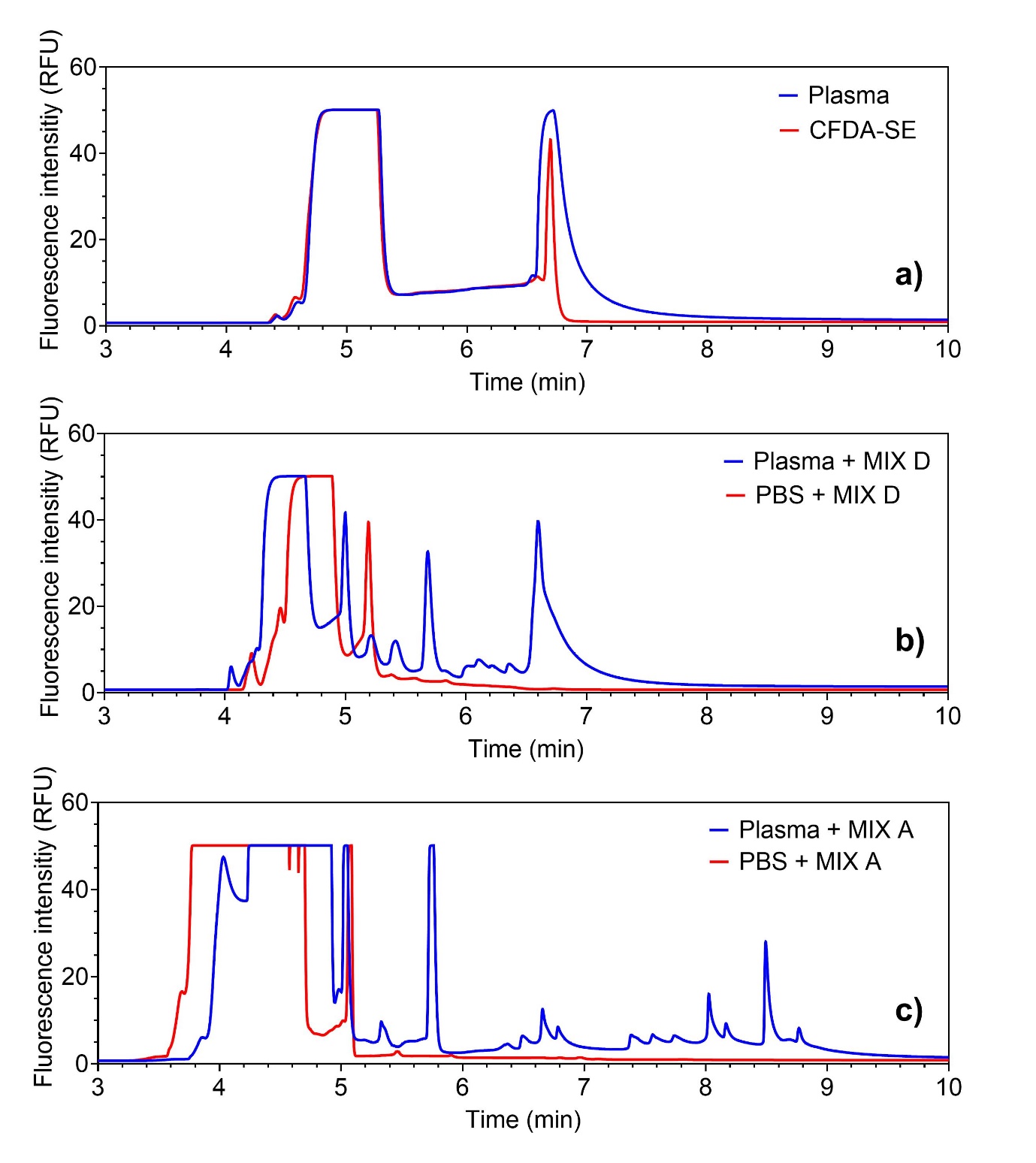


Figure S4. (a) Signal obtained with plasma-derived EVs when no spacer mix is used: the EVs coalesce into a single peak, falling within the same mobility range as one of the CFDA-SE peaks when present alone in solution. (b) In the presence of a different spacer mix containing fewer compounds (MIX D), the same sample shows fewer peaks and less differentiation between subgroups than the signal obtained with MIX A (c).


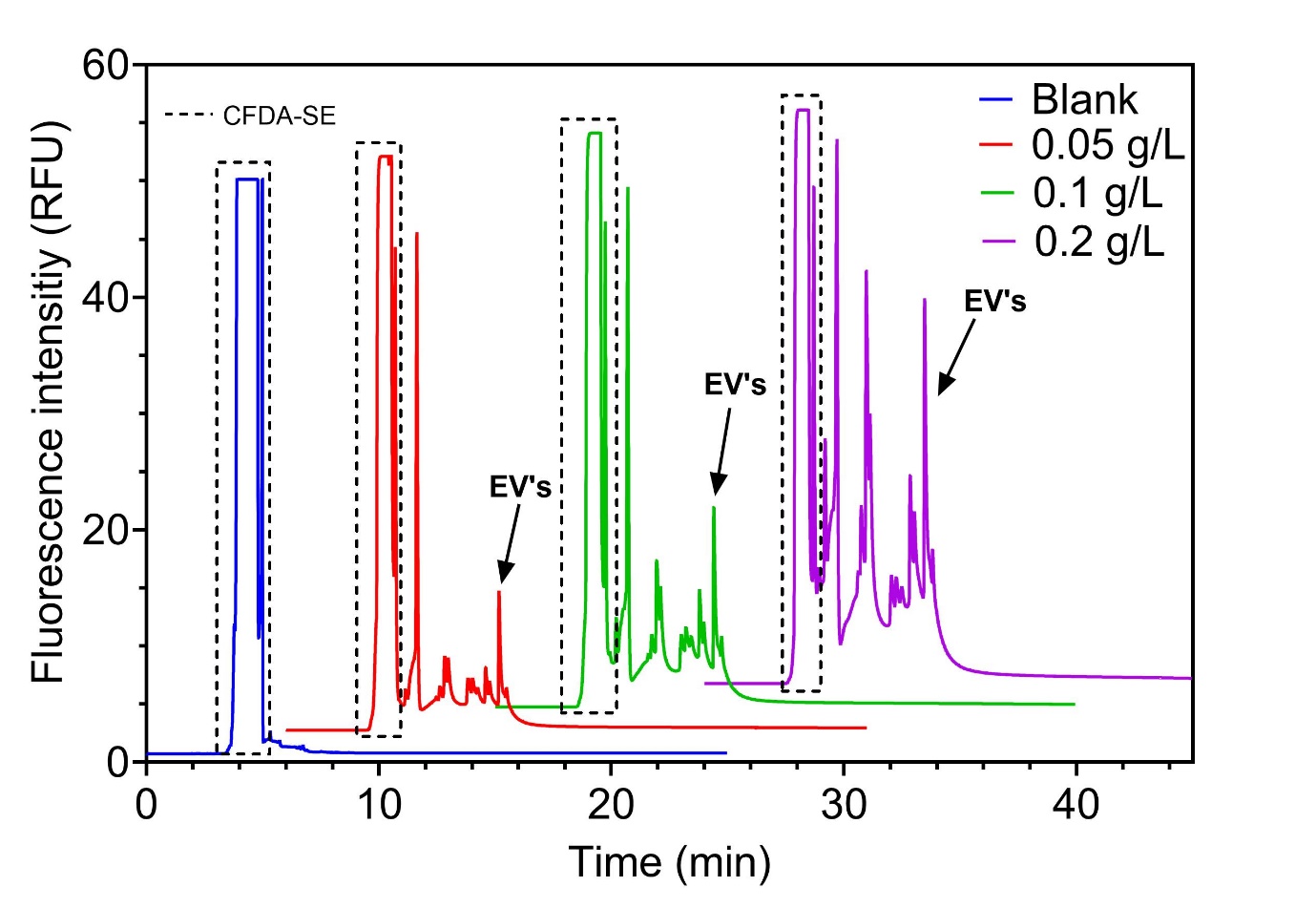


Figure S5. Complete electropherogram of plasma-derived EVs. Three different concentrations of plasma-derived EV samples are tested, and the signal is compared with the one relative to the spacer MIX A alone in the presence of CFDA-SE (in blue). The signal in the EV-related region of the electropherogram (towards lower electrophoretic mobility) scales with the concentration of the EVs. The intense peak at the interface between the dye and the EV areas of the electropherogram can be attributed to contaminants (e.g., protein residues – see Figure S5 or lipoproteins – see Figure 7).


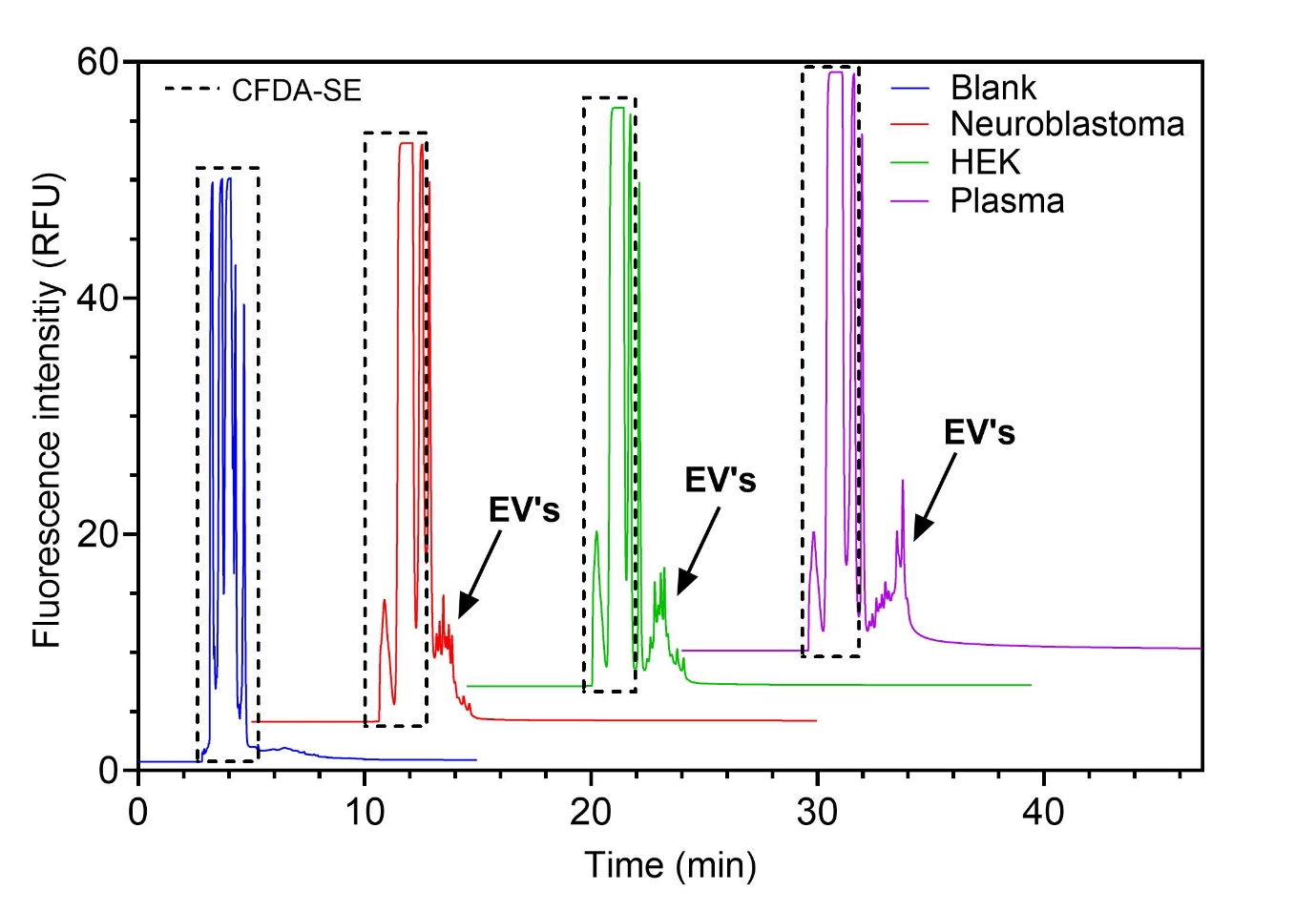


Figure S6. cITP separation of plasma-derived EVs (green), HEK293 cell-derived EVs (red), and SK-N-SH neuroblastoma-derived EVs with the MIX B spacer mixture (in the presence of CFDA-SE, used as a blank). The method can detect distribution differences among EVs derived from various cellular origins.


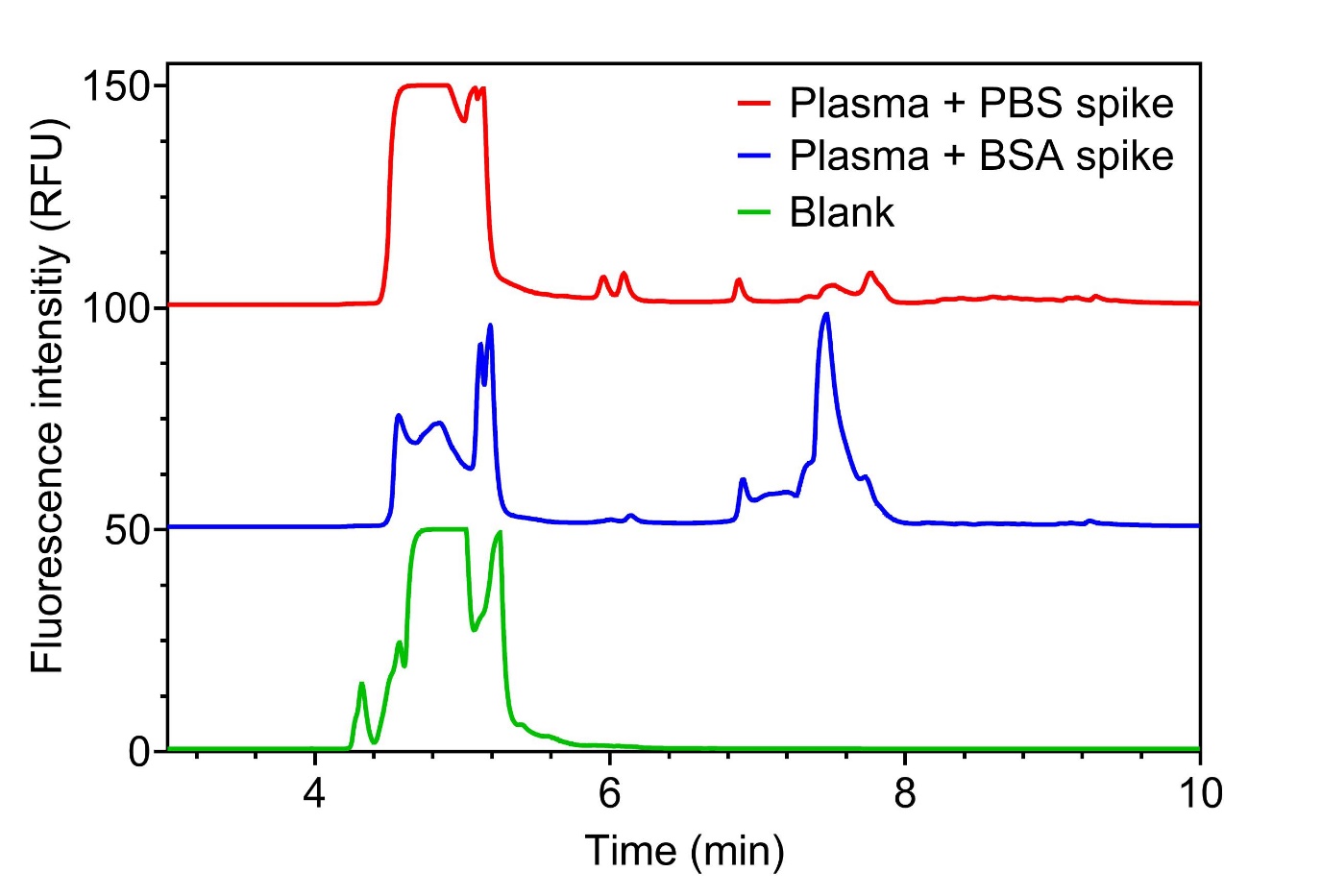


Figure S7. cITP separation of two 0.1 g/L plasma-derived EV samples stained with 10 µM CFDA-SE. One is spiked with PBS at a 1:1 ratio (red), while the other is spiked with a 22.4 mg/l Sigma-Aldrich A2153 BSA solution at a 1:1 ratio (blue). The original spacer mix, made up of 24 compounds, was used for these preliminary measurements, and it is shown as the blank signal. To prevent signal saturation, for this experiment, a lower concentration of CFDA-SE was used, which, although it evidently stained the BSA, resulted in extremely low fluorescence of the peaks related to the EVs (the other experiments presented in the paper have a CFDA-SE concentration of 40 µM).


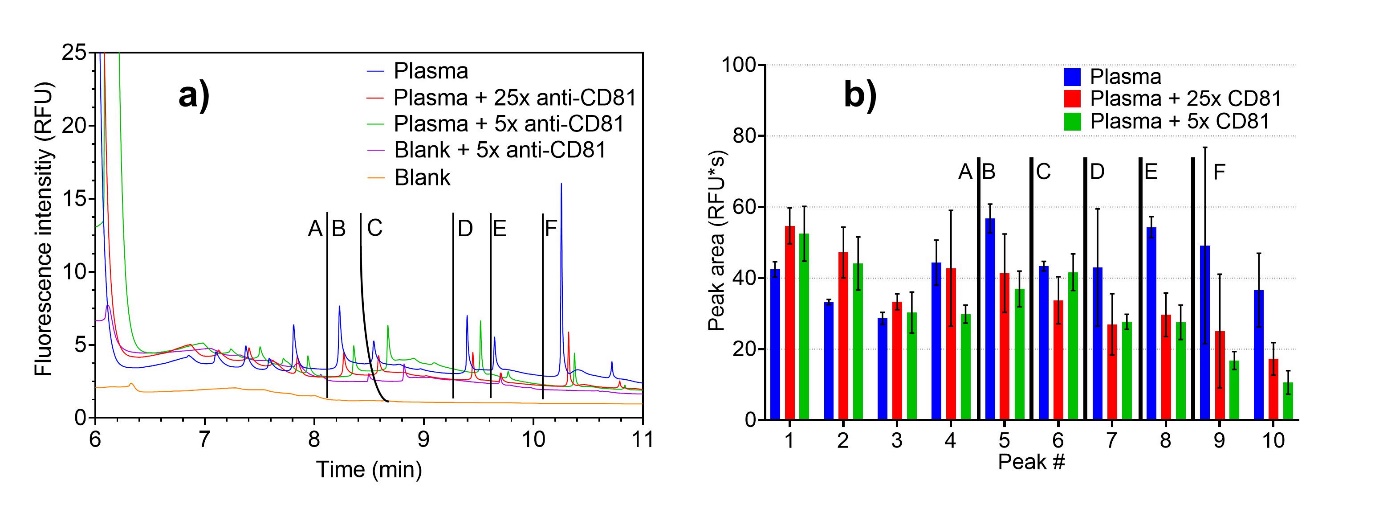


Figure S8. A detailed analysis reveals six distinct zones (A-F), grouping peaks with similar electrophoretic mobility across different samples for easier comparison. Blank: MIX C. The first four peaks show inconsistent effects, with some diminishing and others increasing in intensity as the concentration of anti-CD81 increases (area A). Sections E and F show only peaks that decrease in signal strength with higher anti-CD81 concentration. In sections B, C, and D (peaks 5-7), the fluorescent signal weakens with a 25x dilution of anti-CD81 but strengthens or remains comparable with a 5x dilution, indicating a higher impact of the fluorescent anti-CD81-CFDA-SE complex at higher concentrations. The most impacted peaks are in sections E and F, comprising the slowest-moving particles, indicating the presence of CD81-containing extracellular vesicles. The significant impact of anti-CD81 in areas E and F suggests these peaks contain the purest EV particles, while peaks with less pronounced decreases may include other particle types not expressing CD81.


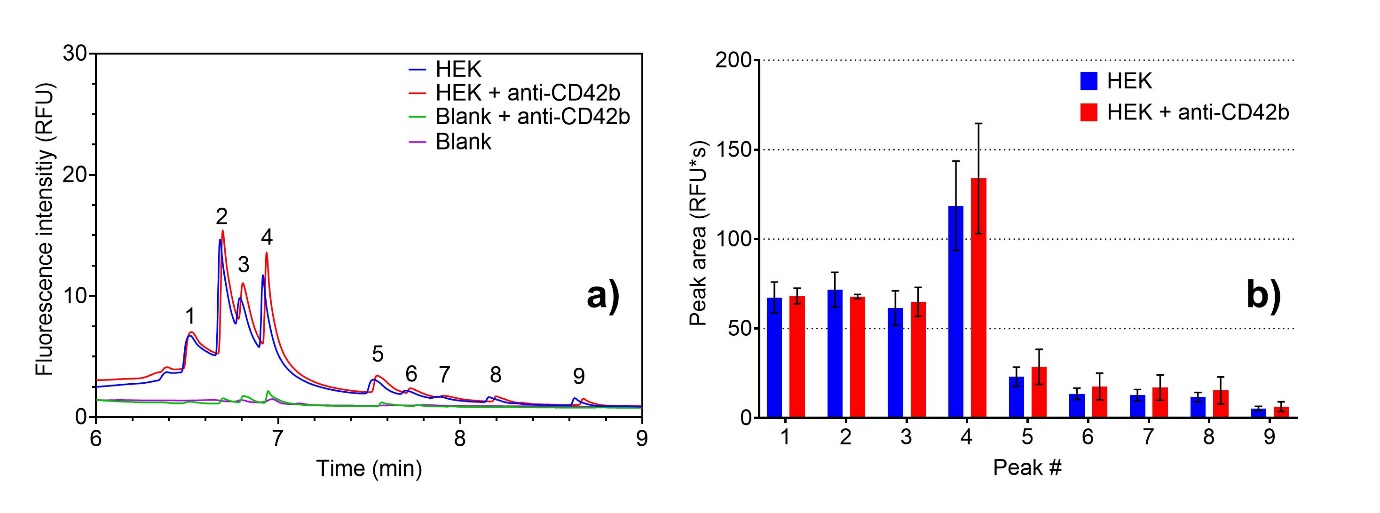


Figure S9. Zoomed-in view of a cITP separation of 0.1 g/L HEK cell-derived EVs in the presence of anti-CD42b antibody and control samples. Green: no EVs (PBS1x) /5x diluted anti-CD42b; blue: HEK EVs/No anti-CD42b; red: HEK EVs/5x diluted anti-CD42b. Blank: MIX B. Average area of peaks 1-10 over 3 different measurements. The standard deviation is presented as an error bar for each peak. As anti-CD42b antibodies target platelets EVs, peak intensity does not decrease in HEK-derived EV samples, unlike with anti-CD81.


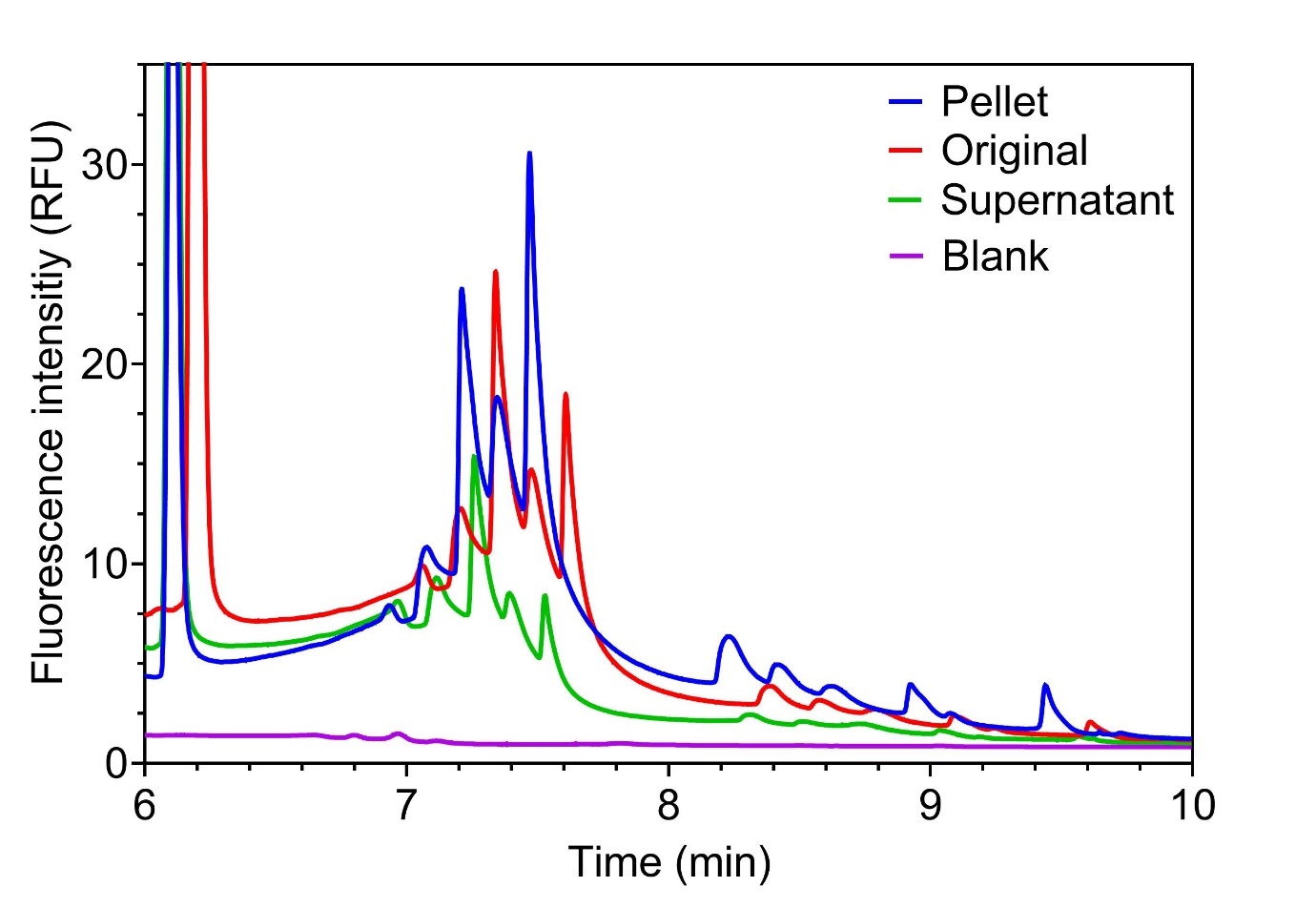


Figure S10. Zoomed-in view of a cITP separation of 0.5 g/L HEK cell-derived EVs before (“Original”) and after a 2 h centrifugation (“Pellet” and “Top fraction”) at 100000 xg (blank: mix A). All pellet peaks show higher intensities than the original sample and supernatant, with a slight temporal shift. Peaks nearly disappear in the supernatant between 8 and 10 minutes, indicating sedimentation into the pellet. Notably, peaks occurring between 8 and 10 minutes nearly disappear in the supernatant, indicating the sedimentation of the analytes into the pellet. The data indicates a general susceptibility of all peaks associated with EVs derived from cell culture to ultracentrifugation, in contrast to plasma-derived EVs. Considering the region of the electropherogram where these peaks are situated (EV-related zone), it is evident that ultracentrifugation effectively sedimented the EVs.

**Supporting information file:**

Table S1 - Spacer mixture compositions.

Figure S1 - TEM images of HansaBiomed SK-N-SH Neuroblastoma-derived EVs (0.5 mg/ml) used for the cITP experiments.

Figure S2 - Dynamic light scattering (DLS) analysis of SK-N-SH neuroblastoma-derived EVs (HansaBiomed, concentration 0.5 mg/ml) used in the cITP experiments.

Figure S3 - Experiment showing the effects of varying the concentration (0.32 mg/ml vs. 2.1 mg/ml) of a specific spacer compound, TAPS, using MIX A and a pre-purified plasma-derived EV sample.

Figure S4 – cITP separation of plasma-derived EV sample when no spacer mix is used and when MIX D is used.

Figure S5 - cITP separation of plasma-derived EV sample at different concentrations.

Figure S6 – Comparison of cITP separation of plasma-derived EV sample, HEK293 cell-derived EVs, and SK-N-SH neuroblastoma-derived EVs.

Figure S7 – Effect of BSA spiking on cITP of plasma-derived EVs.

Figure S8 – Effect of anti-CD81 on cITP of plasma-derived EVs.

Figure S9 - Effect of anti-CD81 on cITP of HEK293 cell-derived EVs.

Figure S10 - Zoomed-in view of a cITP separation HEK cell-derived EVs before and after a 2 h ultracentrifugation experiment.
