# Supplementary figures and images for: Enhanced Separation of Extracellular Vesicles Using Capillary Isotachophoresis With Spacer Compounds
Source: Electrophoresis. 2025 Apr 6;46(9-10):513–23. doi: 10.1002/elps.202400113 (PMC12273895; doi:10.1002/elps.202400113)

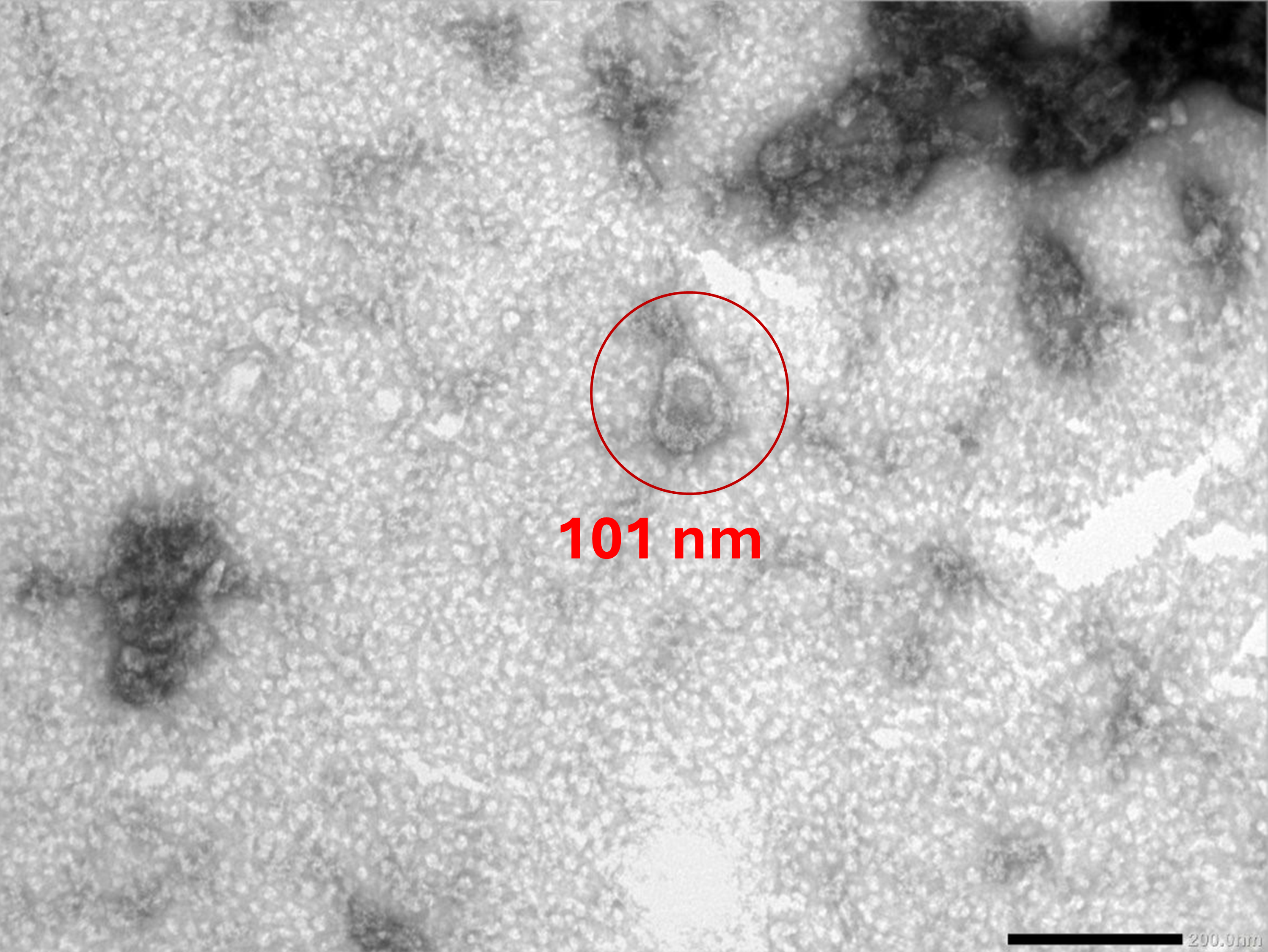

Supplement: Supplementary file 2 — Supporting Information [file ELPS-46--s001.PNG]

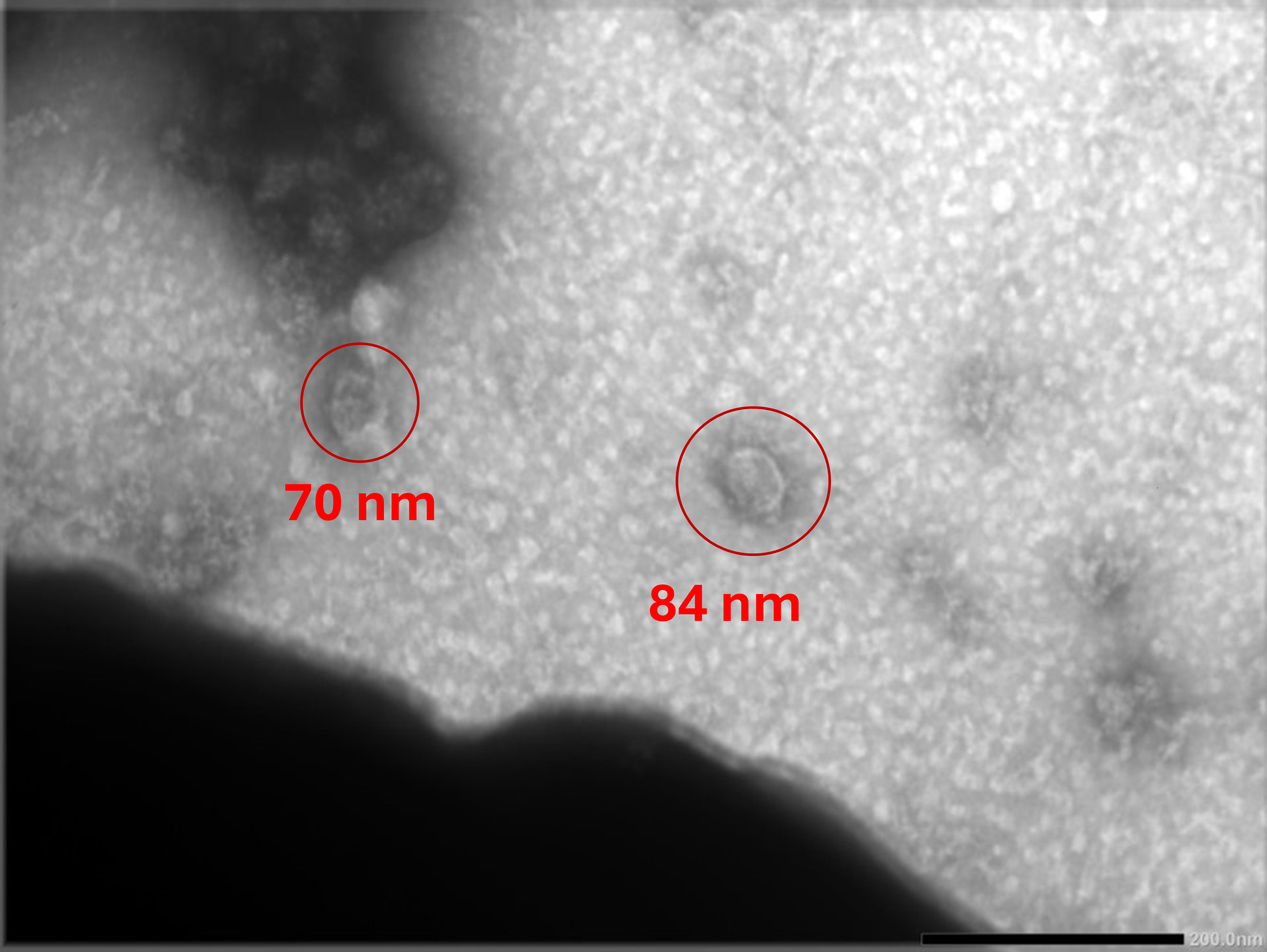

Supplement: Supplementary file 3 — Supporting Information [file ELPS-46--s004.PNG]

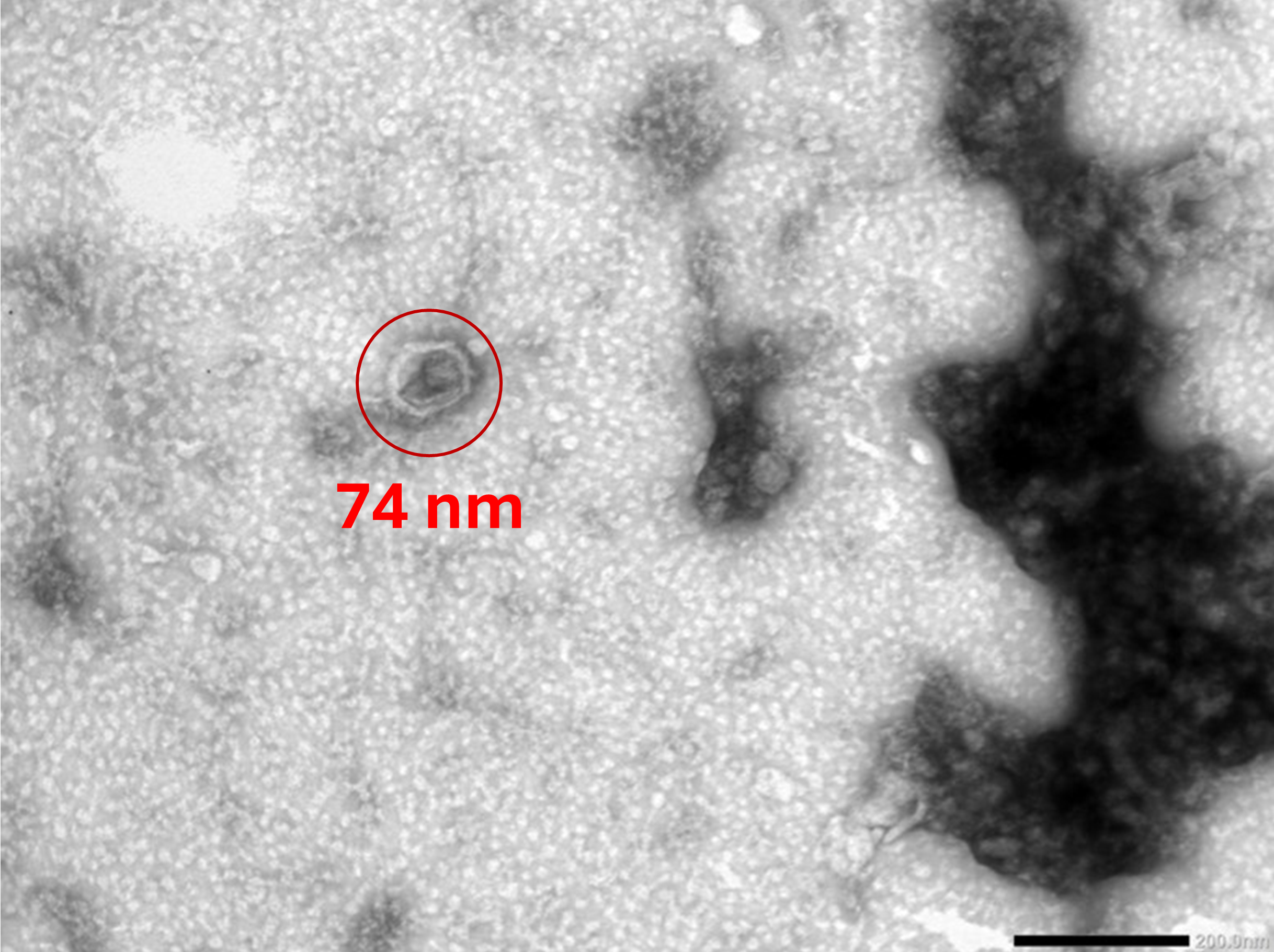

Supplement: Supplementary file 4 — Supporting Information [file ELPS-46--s003.PNG]
